# Molecule-Level Multiscale Design of Nonflammable Gel Polymer Electrolyte to Build Stable SEI/CEI for Lithium Metal Battery

**DOI:** 10.1007/s40820-024-01508-z

**Published:** 2024-09-27

**Authors:** Qiqi Sun, Zelong Gong, Tao Zhang, Jiafeng Li, Xianli Zhu, Ruixiao Zhu, Lingxu Wang, Leyuan Ma, Xuehui Li, Miaofa Yuan, Zhiwei Zhang, Luyuan Zhang, Zhao Qian, Longwei Yin, Rajeev Ahuja, Chengxiang Wang

**Affiliations:** 1 Key Laboratory for Liquid-Solid Structural Evolution and Processing of Materials (Ministry of Education), School of Materials Science and Engineering, Shandong University, Jinan, 250061 People’s Republic of China; 2 Condensed Matter Theory, Department of Physics and Astronomy, Uppsala University, Uppsala, 75120 Sweden

**Keywords:** Anchoring effect, Nonflammable gel electrolyte, In situ cross-linked, Electrode–electrolyte interface, Li metal battery

## Abstract

The risk of flammability is an unavoidable issue for gel polymer electrolytes (GPEs). Usually, flame-retardant solvents are necessary to be used, but most of them would react with anode/cathode easily and cause serious interfacial instability, which is a big challenge for design and application of nonflammable GPEs. Here, a nonflammable GPE (SGPE) is developed by in situ polymerizing trifluoroethyl methacrylate (TFMA) monomers with flame-retardant triethyl phosphate (TEP) solvents and LiTFSI–LiDFOB dual lithium salts. TEP is strongly anchored to PTFMA matrix via polarity interaction between -P = O and -CH_2_CF_3_. It reduces free TEP molecules, which obviously mitigates interfacial reactions, and enhances flame-retardant performance of TEP surprisingly. Anchored TEP molecules are also inhibited in solvation of Li^+^, leading to anion-dominated solvation sheath, which creates inorganic-rich solid electrolyte interface/cathode electrolyte interface layers. Such coordination structure changes Li^+^ transport from sluggish vehicular to fast structural transport, raising ionic conductivity to 1.03 mS cm^−1^ and transfer number to 0.41 at 30 °C. The Li|SGPE|Li cell presents highly reversible Li stripping/plating performance for over 1000 h at 0.1 mA cm^−2^, and 4.2 V LiCoO_2_|SGPE|Li battery delivers high average specific capacity > 120 mAh g^−1^ over 200 cycles. This study paves a new way to make nonflammable GPE that is compatible with Li metal anode.

**Supplementary Information:**

The online version contains supplementary material available at 10.1007/s40820-024-01508-z.

## Highlights

- Nonflammable gel polymer electrolyte (SGPE) is developed by in situ polymerizing trifluoroethyl methacrylate (TFMA) monomers with flame-retardant triethyl phosphate (TEP) solvents and LiTFSI–LiDFOB dual lithium salts.
- Molecular polarity interaction between TEP and PTFMA mitigates interfacial reactions and changes the solvation of Li^+^.
- SGPE forms stable inorganic-rich solid electrolyte interface/cathode electrolyte interface layer, exhibiting well compatibility with Li anode and LiCoO_2_-type high-voltage cathode.

**Supplementary Information:**

The online version contains supplementary material available at 10.1007/s40820-024-01508-z.

## Graphical abstract

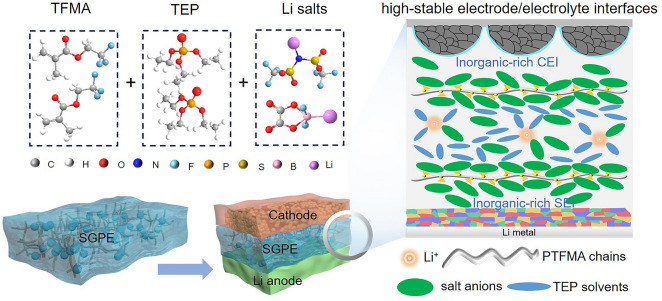

## Introduction

Lithium metal anodes (LMAs) are considered one of the most promising candidate anode materials for high-energy-density power systems because of their ultrahigh specific capacity (3860 mAh g^–1^) and ultralow redox potential (−3.04 V vs Li^+^/Li) [1–3]. Nonetheless, the application of LMAs is limited by notorious safety issues posed by uncontrolled Li dendrite growth with the usage of liquid electrolytes, which results in premature battery failure [4–6]. Recently, in order to suppress the unfavorable dendrites [7, 8] at the Li–electrolyte interface, researches have been intensively developed to obtain a prolonged cycle life, including Li anode modification [9, 10], electrolyte additives [11, 12], developing solid-state electrolytes (SSEs) [13, 14] and other approaches. Generally, SSEs have recently attracted increasing attention due to their high mechanical strength to block Li dendrite penetration [15, 16] and nonleakage properties to avoid electrolyte combustion [17].

Among the SSEs, solid polymer electrolytes (SPEs) stand out benefitting from inherent advantages of light weight, low cost, softness and the flexibility, thus relieving the interface problems caused by poor interfacial contact in cycling to some extent [18–21]. However, most SPEs suffer from inherit high degree of crystallinity and limited Li^+^ conductivity at room temperature; therefore, batteries with SPEs usually need work at a high operating temperature (e.g., at 60 °C) [22–24]. In addition, many SPEs are usually as prepared to be a thin solid film via ex situ casting processes before assembled into batteries, which will result in inferior interfacial wettability with both electrodes, leading to high internal resistance and unfavorable capacity fading [25, 26].

To improve the Li^+^ conductivity of SPEs, gel polymer electrolytes (GPEs) are considered a feasible way by introducing organic liquid electrolytes (LEs) into the polymer matrix [27, 28]. GPEs can integrate superiority of both LEs and SPEs, demonstrating ameliorative ionic conductivity [6, 7, 29] and better nonvolatility [30]. For example, Guo et al. developed a flexible QSPE via in situ polymerization using 1,3,5-trioxane-based precursor and electrolyte, which delivers an excellent ionic conductivity up to 2.5 mS cm^−1^ at 30 °C [6]. Nevertheless, most of introduced liquid moieties in GPEs exhibit poor reductive stability with LMAs, which usually causes irreversible interface corrosion between GPEs and lithium metal [31, 32]. In addition, application of organic solvents is also hindered by their limited oxidative stability once using high-voltage cathodes, resulting in thick cathode electrolyte interphases (CEIs) evolution, cracking of active material particles and transition metals (TMs) dissolution, which significantly destroys the cathode interface and shortens cycle life [5, 33, 34]. Moreover, due to the flammable nature of organic solvents (or plasticizers), GPEs usually show undesirable flammability even at room temperature, dramatically causing the decrease in the safety of the battery [35]. Therefore, it is still a critical challenge to develop a gel polymer electrolyte that integrates high ionic conductivity, improved interfacial compatibility and good thermal stability without making tradeoffs among these properties.

Due to the admirable nonflammability, phosphates solvents are considered as potential plasticizer of GPEs [36, 37]. Among them, triethyl phosphate (TEP) is typical to dissolve various lithium salts and facilitate appropriate ionic conductivity due to high polarity of -P = O groups [36, 38]. However, the poor stability of nomadic TEP molecules on both electrodes usually causes irreversible interface corrosion [33], which severely deteriorates the cycle performance and therefore hinders the further improvement of energy-density of batteries.

To address the above challenge and then expand the TEP-based GPE into a high-performance electrolyte, an elaborated GPE is designed and synthesized in this work via in situ polymerization process by systematically adjusting the relative concentration of the monomer (trifluoroethyl methacrylate, TFMA), solvent (triethyl phosphate, TEP) and Li salts (lithium bis(trifluoromethanesulfonyl)imide, LiTFSI and lithium difluoro(oxalato)borate, LiDFOB). Further novelties are considered in this process. Firstly, each TFMA monomer contains three C-F groups, which increases the polarity of polymer chains and helps effectively capture TEP molecule through intermolecular interactions, thus restricting the TEP migration and weakening side interactions. An anion-dominated Li^+^ solvation structure is constructed, which induces anion-derived solid electrolyte interface/cathode electrolyte interface (SEI/CEI) protective layer and fast Li^+^ structural transport. For comparison, methyl ethyl acrylate (HEMA, Fig. S1) without polar groups is also employed as the polymer matrix of GPE. The comparative study subsequently reveals that the polymer matrix without high-polarity functional groups could only act as a cosolvent, increasing the content of free TEP and thus exacerbating the side reactions of the electrodes. Secondly, density functional theory (DFT) calculations reveal that the introduction of F contained functional groups into ester polymer monomer is proposed to improve the oxidation existence and build a fluorine-rich interface layer between electrolyte and anode at the same time [39], which significantly guarantees the stability of the high-energy-density electrode–electrolyte interface. Thirdly, to further reinforce the electrode–electrolyte interface, LiTFSI–LiDFOB dual lithium salts are used due to the high dissociation ability of LiTFSI and the excellent film-forming property of LiDFOB [40, 41]. Finally, the in situ cross-linking strategy of the TFMA-containing precursor enables the electrolyte with robust wettability on electrode surface, dramatically decreasing the interfacial resistance caused by poor interfacial contact. These electrochemically optimized strategies enable fast lithium-ion transport kinetics with high conductivity (1.03 mS cm^–1^ at 30 °C), a higher Li^+^ transference number (0.41) and excellent lithium plating/stripping reversibility on a lithium anode. It turns out that LiFePO_4_-based full cells with our designed electrolyte can achieve stable cycling more than 500 cycles under 0.5 C at room temperature, which is mainly ensured by the TEP-anchoring effect of TFMA. This rationally designed GPE with anchoring effect can also be applied to other types of batteries.

## Experimental Section

### Materials

TFMA (98%) monomer and 2,2'-azobis(2-methylpropionitrile) (AIBN, 99.5%,) was supplied by Alladin Co., Ltd. (China). Lithium bis(trifluoromethanesulfonyl)imide (LiTFSI, 99.9%) salt, lithium difluoro(oxalato)borate (LiDFOB, 99.9%) salt and triethyl phosphate (TEP, 99.8%) solvent were purchased from DuoDuo Chem Co., Ltd. (China).

### Preparation of Different TEP-Based Electrolytes and Battery Fabrication

#### Preparation of Different TEP-Based Electrolytes

Highly stable GPEs were prepared by the mixing and in situ polymerization of TFMA (98%) monomer, lithium bis(trifluoromethanesulfonyl)imide (LiTFSI, 99.9%) salt, lithium difluoro(oxalato)borate (LiDFOB, 99.9%) salt, triethyl phosphate (TEP, 99.8%) solvent and 2,2′-azobis(2-methylpropionitrile) (AIBN, 99.5%). The proportions of TEP, LiTFSI and TFMA were systematically varied, while the concentration of LiDFOB was kept constant. TEP was mixed with TFMA monomer at different volumetric ratio (35:65, 40:60, 60:40, 80:20); then, LiTFSI and LiDFOB were added to the above system based on different concentrations (0.2, 0.5, 1, 1.5 and 2 M) and a constant concentration of 0.5 M, respectively. After that, 1 wt% 2,2′-azobis(2-methylpropionitrile) (AIBN, 99.5%) as an initiator was added to the precursor and mixed thoroughly to obtain the precursor solution. Then, 50.0 µL of the as obtained homogeneous precursor was injected into cellulose separator and the cell was immediately assembled and then heated at 60 °C for 12 h for complete polymerization. The samples were denoted as “xTFMA-yLF-0.5LB,” according to their LiTFSI concentrations and TFMA polymer precursor content. Taking the example of 60TFMA-0.2LF-0.5LB system, it represents a GPE electrolyte that contains 60 vol% of TFMA (TFMA% = V_TFMA_/(V_TFMA_ + V_TEP_)) monomer in the precursor mixing with 0.2 M LiTFSI and 0.5 M LiDFOB. Subsequently, the 35TFMA-0.5LF-0.5LB sample was abbreviated SGPE. The comparative sample CGPE was prepared by replacing TFMA with an equal volume of HEMA in SGPE, and then, a homogeneous gel was also obtained under the same reacting condition. As for the comparative LE group, it can be prepared by dissolving 0.5 M LiDFOB and 0.5 M LiTFSI salts in the pure TEP electrolyte. All of the above procedures were performed inside an argon-filled glove box with moisture/oxygen concentrations below 0.01 ppm.

#### Preparation of Samples for the Verification (FTIR and Raman Characterization) of Anchoring Effect

The samples for FTIR characterization were prepared by mixing TEP with TFMA in volumetric ratios of 35:65, 40:60, 60:40 and 80:20 and then stirred until mixed evenly. After initiated by 1% mass of AIBN and then heated at 60 °C for 12 h, the finally obtained samples were named 35TFMA-65TEP, 40TFMA-60TEP, 60TFMA-40TEP and 80TFMA-20TEP according to gradient volumetric fraction in the corresponding samples. Samples of 35HEMA-65TEP, 40HEMA-60TEP, 60HEMA-40TEP and 80HEMA-20TEP can be obtained by replacing TFMA with HEMA under the same synthetizing process. The samples for Raman characterization were prepared by mixing TEP with TFMA and HEMA polymer monomers under a volumetric ratio of 65:35, respectively. Then, 1 M LiTFSI and 1% AIBN were added to the above solution and a transparent precursor solution can be obtained after being stirred evenly. The final samples can be obtained after heating at 60 °C for 12 h and were labeled as 1 M-SGPE and 1 M-CGPE, respectively. All of the above sample preparation procedures were conducted in an argon-filled glove box with moisture/oxygen concentrations below 0.01 ppm.

#### Electrode Preparation and Battery Fabrication

The LFP and 4.2 V LCO cathode were prepared by a simple slurry coating method. The active cathode material, super P and polyvinylidene fluoride (PVDF) binder were grinded together in a mass ratio of 80:10:10 in anhydrous N-methyl-2-pyrrolidone (NMP, 99.9%, MTI). The slurry was magnetically stirred for 8 h and then coated on an aluminum current collector and dried overnight at 80 °C. After that, the LFP-based cathode was punched into disks with a common mass loading of 1.5–2.0 mg cm^−2^ LFP. The 4.2 V LCO-based cathode with a mass loading of 1–1.5 mg cm^−2^ active materials loading can also be prepared following the same procedure. Subsequently, coin cells (CR2025 or CR2032 type) were assembled with Li foil working electrodes (with a thickness of 45 μm), Li foil or LFP/4.2 V LCO counter electrodes, NKK-TF4030 cellulose separator and the corresponding electrolyte precursor (50 μL) and then heating at 60 °C for 12 h to complete the polymerization. All batteries were assembled in an argon-filled glovebox with less than 0.1 ppm O_2_ and 0.1 ppm H_2_O.

### Electrochemical Characterizations

Electrochemical measurements were taken by using CR2025 coin cells or CR2032 coin cells. An electrochemical workstation ((CHI600E) was used for electrochemical impedance spectroscopy (EIS), lithium-ion transference number (t_Li+_) and linear sweep voltammetry (LSV).

Ionic conductivities of different gel electrolyte systems at varied temperatures ranging from 30 to 80 °C were measured by EIS. The corresponding precursor solution was infiltrated into porous cellulose membrane sandwiched between two stainless steel (SS) plate electrodes in a SS|GPE|SS configuration. The ionic conductivity, σ, was calculated depending on Eq. (1):

1 $$\upsigma =\frac{L}{RS}$$

where *L* is the thickness of the GPE electrolyte, *S* is the contact area between GPEs and SS electrode and *R* is the resistance measured from EIS in the frequency ranging from 0.1 Hz to 10 MHz with an AC amplitude of 10 mV. To investigate the temperature-dependent ionic conductivity for GPEs, the activation energy (*E*_a_) was evaluated according to Arrhenius Eq. (2):

2 $${\sigma }_{T}=A{e}^{\frac{{-E}_{a}}{{k}_{B}T}}$$

where A is a pre-exponential factor, *E*_*a*_ is activation energy, *k*_*B*_ is the Boltzmann constant and *T* is the absolute temperature. The Li^+^ transference number, t_Li+_, was determined by a combined method of chronoamperometry and AC impedance spectroscopy with a Li||Li symmetric cell, according to Eq. (3):

3 $${t}_{{Li}^{+}}=\frac{{I}_{SS}(\Delta V-{I}_{0}{R}_{0})}{{I}_{0}(\Delta V-{I}_{S}{R}_{S})}$$

where* I*_0_ and *I*_ss_ are the initial and steady-state currents measured by chronoamperometry under a small polarization potential (10 mV), and *R*_0_ and *R*_ss_ are the resistances of the symmetric cell before and after polarization. LSV was studied between 1.0 and 6.0 V versus Li/Li^+^ at a scan rate of 10 mV s^−1^ with a Li|SS asymmetric cell to measure the electrochemical window.

The galvanostatic charging–discharging of battery cells was performed in LAND CT2001A. As for symmetrical Li cells, the current density is 0.1 mA cm^−2^. 4.2 V LCO||Li cells and LFP||Li cells were cycled with cutoff voltages of 3–4.2 and 2.5–4 V, respectively.

### Materials Characterization

X-ray diffraction (XRD) patterns were collected on Rigaku D/Max-KA X-ray diffractometer equipped with a Cu Kα source (λ = 1.5406 Å). The surface or fracture section morphologies of the SGPE electrolyte, deposited Li and cycled LCO particles were characterized by field emission scanning electron microscopy (FESEM, SU-70), with energy-dispersive spectrometer (EDS) to characterize the elemental distributions of the materials. Fourier transform infrared spectroscopy (FTIR) tests by Bruker spectrometer TENSOR 27 and ^1^H/^19^F NMR analysis by Bruker AVANCE III HD 600 were carried out to character the radical polymerization reaction of TFMA/HEMA monomers and the anchoring effect in SGPE system. Raman spectra investigated by HORIBA LabRAM HR800 Raman spectrometer are further performed to figure out the coordination structure under the influence of anchoring effect. Thermal characteristics of different electrolytes were evaluated by thermogravimetric analysis (TGA, TGA8000-SQ) and differential scanning calorimetry (DSC, Netzsch-3500) under the protective N_2_ atmosphere at a heating rate of 10 °C min^−1^.

To characterize the crystalline structure of cycled LiCoO_2_ particles from different electrolyte systems, TEM images were acquired using a Hitachi HT-7700 transmission electron microscope. Before experimenting, the LiCoO_2_ particles were thoroughly washed with dimethyl carbonate (DMC) for several times to remove the residual electrolytes. The composition on the cycled electrodes surface or in different depths was further employed by high-resolution X-ray photoelectron spectroscopy (XPS) recorded by Thermo SCIENTIFIC ESCALAB Xi^+^ instrument equipped with a 1486.68 eV Al Kα probe beam. These electrodes were rinsed with DMC to remove residual electrolytes and then dried in an argon-filled glove box.

### DFT Calculations

All DFT calculations were carried out with the Gaussian 16 software. The optimized geometric configurations for each molecule were determined by the 6–31 + G (d, p) basis set. The B3LYP functional was adopted in calculations. The binding energy (*E*) was calculated by Eq. (4):

4 $$E={E}_{P+T}-{E}_{P}-{E}_{T}$$

where *E*_*P+T*_ is the total energy of TFMA, HEMA and TEP with TEP solvents, *E*_*P*_ is the energy of TFMA, HEMA and TEP, and *E*_*T*_ is the energy of TEP. The values of molecular orbits were obtained after structure optimizations.

## Results and Discussion

### Preparation and Electrochemical Characterization of GPEs

**Fig. 1:**
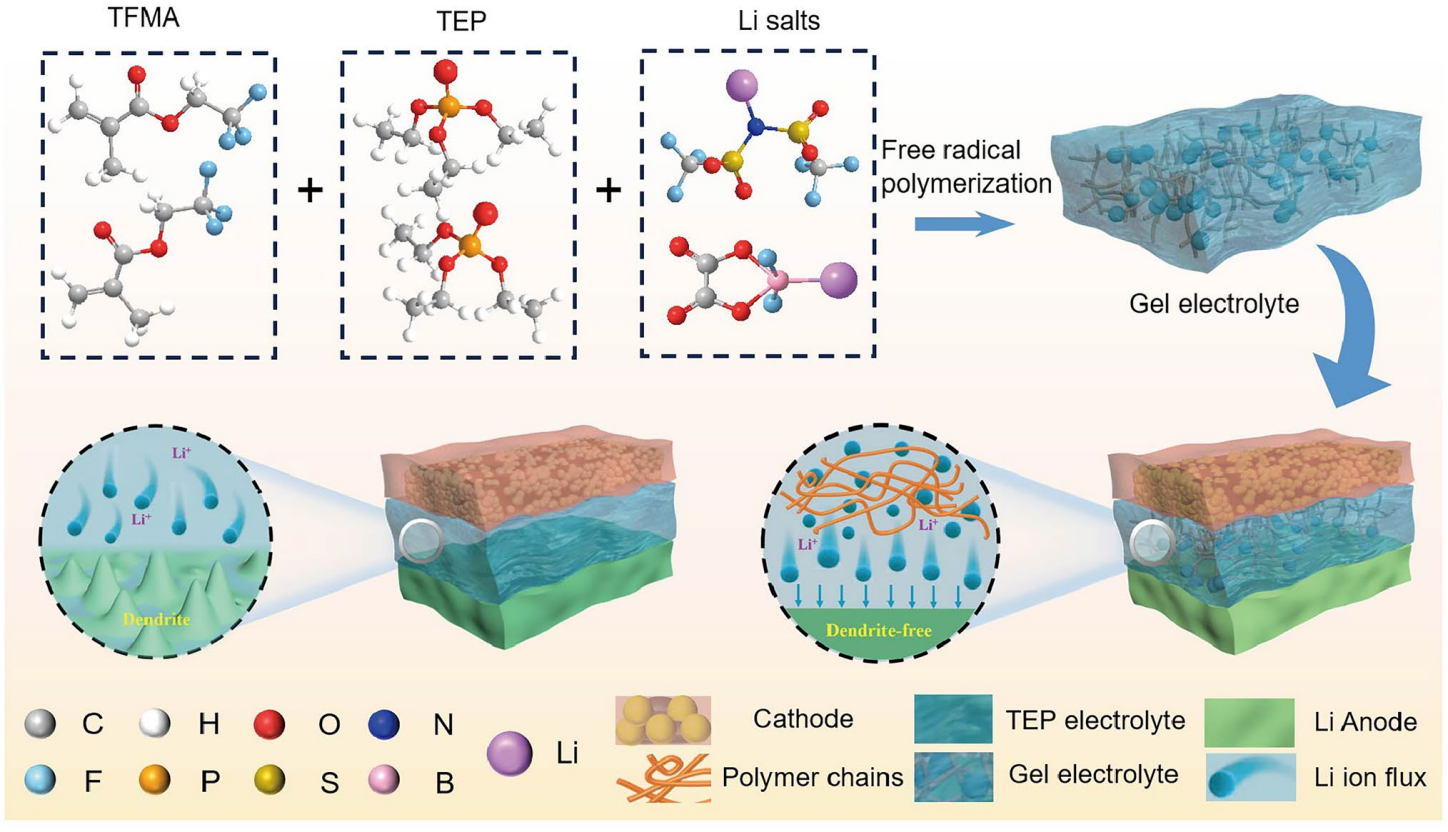
Schematic illustration of in situ preparation of GPE and the difference on Li^+^ transport between the designed gel electrolyte and TEP electrolyte

Schematic of the synthesis process for our designed GPE is shown in Fig. 1. The precursor solution is composed of TFMA monomer, TEP solvent as well as the dual lithium salt, and then, the final GPE can be easily fabricated by the in situ free radical polymerization of (TFMA) initiated by azobisisobutyronitrile (AIBN) under 60 °C for 12 h. In contrast to the TEP-based liquid electrolyte, our designed GPE boosts highly homogenious Li ion flux, leading to more stable Li plating/stripping during cycling process. Optical photographs of the GPE in Fig. S2 show that the polymerized precursor solution is solid-state-like, confirming that TEP is completely confined in the solid phase, and therefore, the leakage of the liquid TEP electrolyte can be reduced to some extent. The morphology of cellulose separator before and after polymerization was explored by SEM and corresponding element mapping were explored (Fig. S3). Original cellulose separator is full of pores ~ 0.5–2 μm, which increases the mechanical strength of gel electrolyte. Obviously, after polymerization of TFMA, the pores are filled with GPE and all elements are uniformly distributed in the membrane, ensuring a homogeneous distribution of Li ions.

**Fig. 2:**
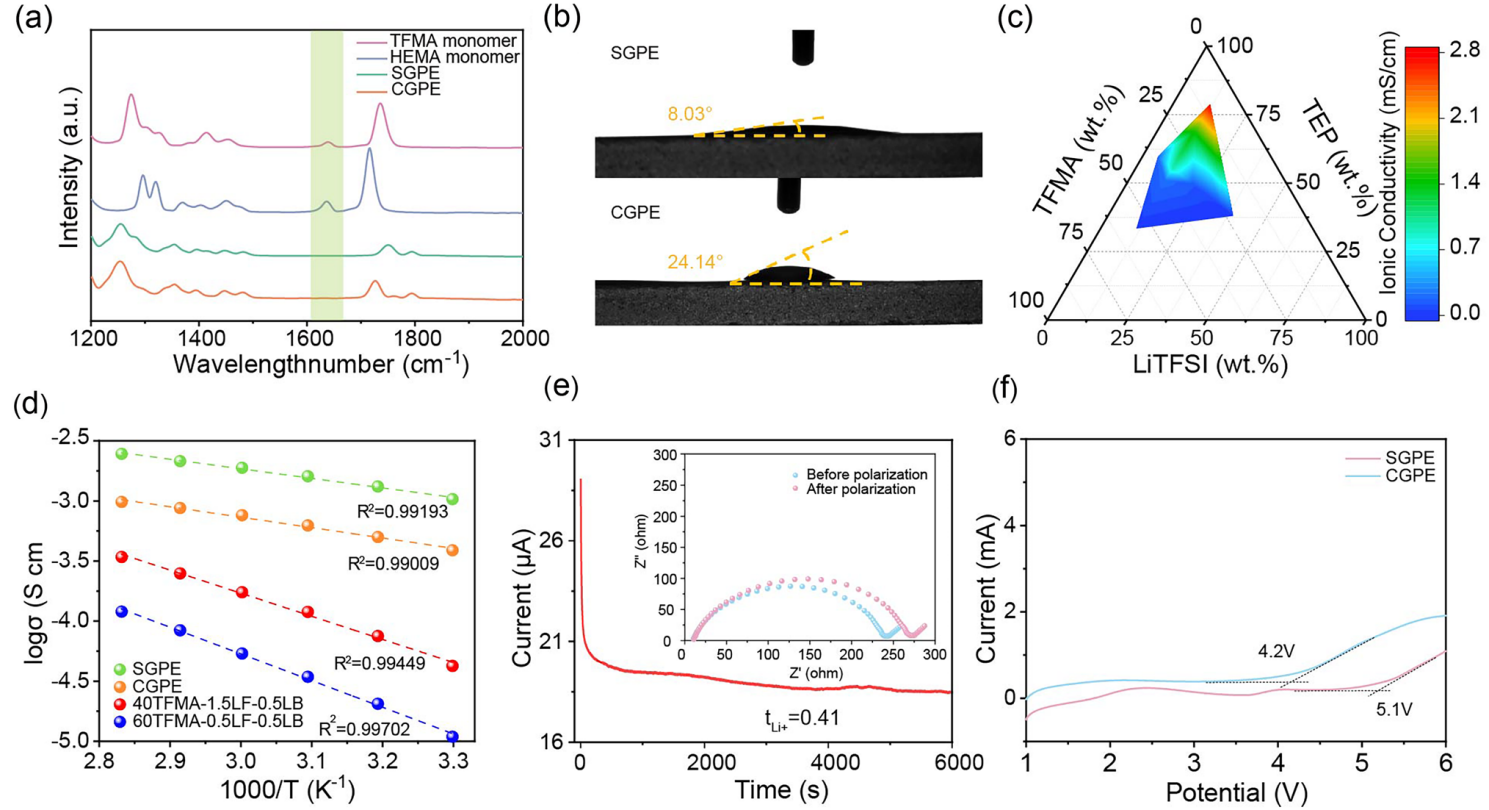
**a** FTIR spectra of TFMA monomer, HEMA monomer and GPEs after polymerization. The marked peaks in green region come from vibration of C = C in TFMA or HEMA monomer. **b** Contact angle of SGPE and CGPE precursor. **c** Ionic conductivity gradient of the TEP-based electrolytes at room temperature on the ternary phase diagram. **d** Ionic conductivities as a function of temperature for the GPEs. **e** Chronoamperometry profile collected from a symmetric Li|SGPE|Li cell (The inset corresponds to the EIS plots before and after chronoamperometry). **f** Linear sweep voltammetry profiles of the Li|SGPE|SS cell and Li|CGPE|SS cell (10 mV s^–1^)

The polymerization process was further tested by Fourier transform infrared spectroscopy (FTIR). As shown in Fig. 2a, disappearance of the -C = C- peak (1635 cm^–1^) belonging to unpolymerized TFMA or HEMA monomer after the heating process indicates complete polymerization. Additionally, the signals at about 1270 ~ 1320, 1260, 1400 ~ 1500 and 1730 ~ 1750 cm^−1^ in spectra of HEMA/TFMA monomer, TEP, monomers/GPEs are belonged to the vibration of -C-F_x_ or –C-O-C, -P = O, -C-H and -C = O groups. After polymerization, the peaks of -C-F_x_ or –C-O-C have been merged in the wide peak of -P = O at ~ 1260 cm^−1^ and the intensity of -C = O also decreases in GPEs, because TEP accounts for a large volume fraction of 65% than that of polymer chains. It has also been found that the stretching vibration peaks for -C-H group in GPEs deliver an obvious shift compared to their monomers, indicating that the polymerization process changes the surrounding environments of -C-H due to the newly extended polymer backbone [39, 42–45]. ^1^H NMR spectroscopy in Fig. S4 further confines the structural evolution of the monomers, where broad ^1^H NMR chemical shift appeared after the reaction, implying the formation of polymer matrix [46]. Considering the critical role of wettability of GPEs on cellulose separator, contact angles was measured. As shown in Fig. 2b, the contact angle of comparative CGPE precursor is significantly larger than that of our designed SGPE precursor, indicating that SGPE delivers better wettable interface with cellulose membrane.

To optimize ionic conductivity of TFMA-based gel electrolyte, the ratios of LiTFSI, TEP and TFMA were systematically investigated, while the concentration of LiDFOB was fixed at 0.5 M. The electrolytes are donated as xTFMA-yLF-0.5LB and their compositions are given in Table S1 (Supporting Information), where xTFMA-yLF-0.5LB refers to x vol% of TFMA (x = 35, 40, 60 and 80; TFMA% = V_TFMA_/(V_TFMA_ + V_TEP_)), yM LiTFSI (y = 0.2, 0.5, 1, 1.5, 2 and 2.5) and 0.5 M LiDFOB. Electrochemical impedance spectroscopy (EIS) was used to measure the RT ionic conductivity of the TFMA-based GPEs in Fig. S5, and the calculated index according to Eq. (1) is shown in Table S1. Figure 2c displays ionic conductivity of the TFMA-based GPEs with different ratios of TFMA, TEP and LiTFSI. The ionic conductivity is found to be higher at moderate LiTFSI concentrations (e.g., 0.5 M) and lower polymer matrix content. However, the nonflowing gel is hard to build under low polymer contents like 10%, 20% and 30% (Fig. S2). Therefore, according to Figs. 1c and S2, the balanced polymer content for the consideration of both mechanical stability and ionic conductivity should be 35 vol%. Since it shows exceptional high ionic conductivity (0.93 mS cm^−1^ at RT), 35TFMA-0.5LF-0.5LB GPE was selected for subsequent tests and abbreviated SGPE. Comparatively, the ionic conductivity of 35HEMA-0.5LF-0.5LB GPE electrolyte is calculated to be 0.37 mS cm^−1^ and the electrolyte is denoted as CGPE.

The EIS spectra at different temperature were measured to reveal the relationship between ionic conductivity and temperature in Fig. S6a–d. According to Fig. S6, SGPE exhibits high ionic conductivity of 1.03 mS cm^−1^ at 30 °C, which is much higher than that of CGPE (0.39 mS cm^−1^). Ionic conductivities increase with temperature because of the promotion of Li^+^ movement at high temperature. Temperature-dependent ionic conductivities of GPEs in different compositions are presented in Fig. 2d, whose fitting results follow Arrhenius behavior. The activation energy (*E*_a_) of SGEP is figured out to be 0.16 eV based on this model. Such low *E*_a_ is much better than other GPEs with high contents of TFMA matrix or with HEMA matrix, indicating the fast lithium-ion transport kinetics in SGPE [25, 33].

Another critical parameter for electrolytes is Li^+^ transference number (*t*_Li+_). The *t*_Li+_ of SGPE (Fig. 2e) is calculated up to 0.41, which largely surpasses CGPE (0.11, Fig. S7). Such a high *t*_Li+_ of SGPE can primarily be attributed to the effectively inhibited migration of anions in the polymer matrix, resulting in suppressed dendrite formation [47, 48]. To reveal the oxidative stability of GPEs, electrochemical window based on linear sweep voltammetry (LSV) tests was investigated (Fig. 2f). The SGPE has an obviously higher initial oxidation potential of 5.1 V, while that of CGPE is only 4.2 V. Therefore, SGPE is more stable to match high-voltage cathodes to develop high-energy-density systems. Accordingly, the enhanced oxidation stability may partly benefit from the coordinated polymer skeleton and TEP ligand molecules [49]. When electrolyte reacts with the electrodes during the charging and discharging process, more energy is needed to break this intermolecular interaction, thus leading to increased electrochemical stability [7]. Note that obvious fluctuations between 1.5 ~ 2.5 V can be seen, which can be attributed to the decomposition of LiDFOB [12]. Generally, such a decomposition is beneficial to improve interface stability by introducing inorganic ingredients in CEI layer and thus facilitating fast lithium-ion transport.

### Physical and Cycle Stability Characterization

**Fig. 3:**
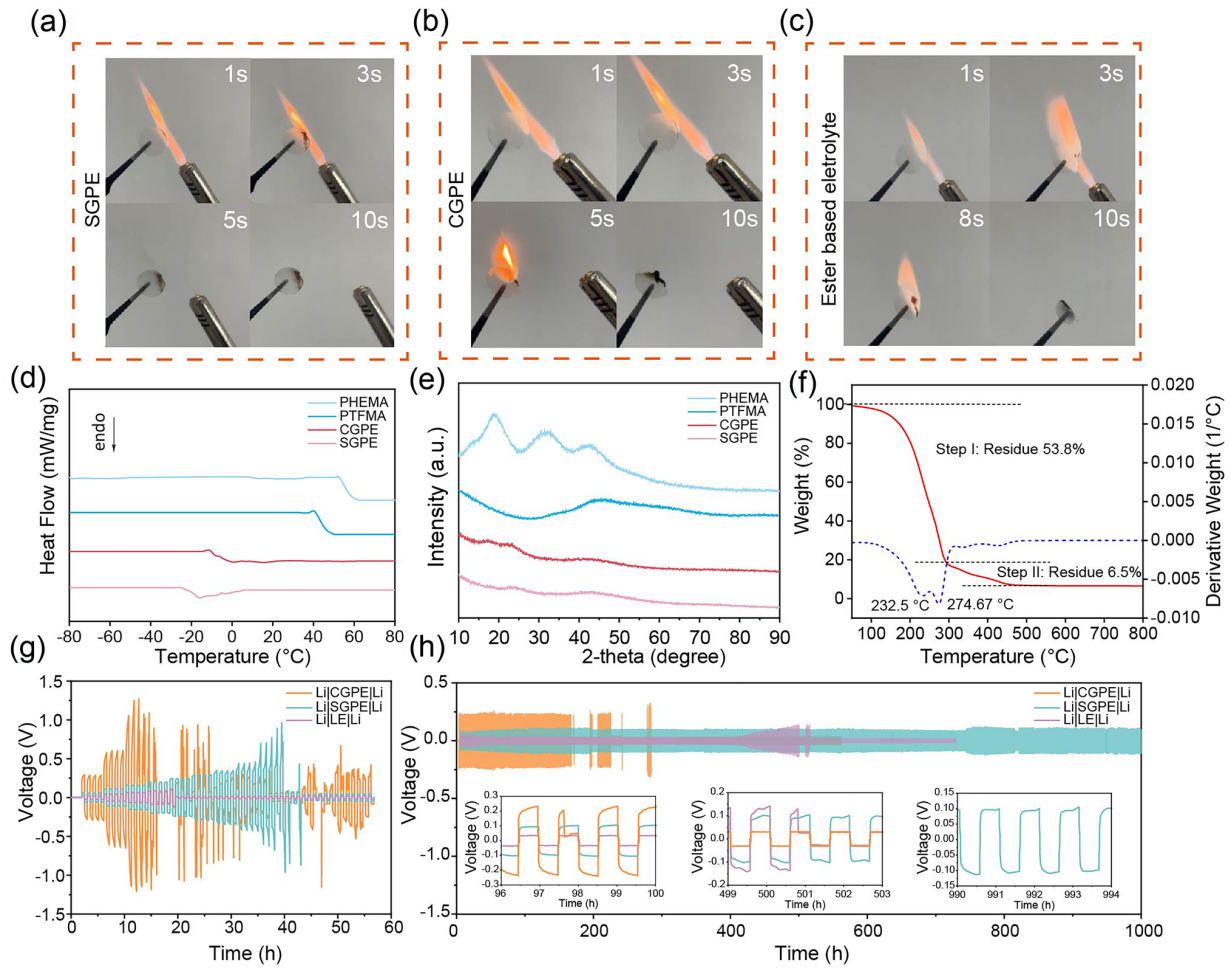
Flammability test of **a** SGPE, **b** CGPE and **c** 1 M LiPF_6_ in EC:DEC:DMC (1:1:1) electrolyte. **d** DSC analysis of the GPEs and pure polymer matrix. **e** XRD patterns of the pure polymer matrix and corresponding gel electrolytes. **f** TGA thermograms of SGPE. **g** Critical current density test of the Li||Li symmetric cells with different electrolytes. **h** Long-term cycling performance of Li||Li symmetrical cells, with a current density of 0.1 mA cm^−2^ and 0.05 mAh cm^−2^ Li plated and stripped per cycle

The thermal stability and flexibility of GPEs are also critical for practical applications. In the flammability test shown in Fig. 3a–c, SGPE demonstrates prompt self-extinguishing behavior after removing fire, which is in sharp contrast to the fierce combustion of CGPE and commercial carbonate electrolytes. Therefore, these results demonstrate the favorable nonflammability and thermal stability of SGPE. DSC was used to investigate the glass transition temperature (*T*_g_) of GPEs and the corresponding polymer matrix. Figure 3d shows that the *T*_g_ of SGPE is −19 °C, which is much lower than pure PTFMA polymer matrix, indicating a much intensive mobility of the polymer segments in the presence of TEP. What is more, compared to the pure polymer matrix, no exothermic recrystallization peak is observed in GPEs, suggesting that GPEs is likely amorphous at ambient temperature. The same conclusion is demonstrated by XRD patterns, which reveal the change from crystalline pure polymer to amorphous gel electrolytes (Fig. 3e). The wide amorphous peaks of GPEs reveal the disordered structure of polymer chains, which agrees well with DSC analysis. Therefore, the Li^+^ could also be conducted through the motion of polymer chains in the GPEs, which is beneficial to improve the Li^+^ conductivity [7]. According to the thermogravimetric (TG) curves in Fig. 3f, the weight loss process of SGPE can be divided into two parts at 275 and 344 °C, where the first weight loss is mainly contributed to the evaporation of liquid-phase TEP [37]. For comparison, CGPE shows significant weight loss at much lower temperature (232 °C) for the first stage (Fig. S8). Hence, it is reasonable to consider that a stronger intermolecular interaction between TEP and PTFMA exists in SGPE, thereby leading to a much-extended volatile range of liquid phase [7]. As shown in Fig. S9, stress–strain tests were also conducted to analyze the mechanical strength of SGPE and CGPE. The GPEs deliver excellent toughness, with fracture elongation of 119.9% and 110.7%, respectively, which greatly contributes to depress dendrite penetration and prolong cycle life.

The gradually increasing current density during long-period cycle will enlarge the potential interfacial side reactions of Li||Li symmetric cells; therefore, it is important to test the critical current density (CCD) of electrolytes to LMAs. The current density gradiently increased 0.05 mA cm^−2^ every 4 cycles from initial state of 0.05 mA cm^−2^. Unsurprisingly, in Fig. 3g, the Li|SGPE|Li symmetric cell shows critical current density up to 0.4 mA cm^−2^ with inconspicuous voltage fluctuations during the CCD test at room temperature. In sharp contrast, the voltage of Li|CGPE|Li symmetric cell fluctuates sharply when the current density increases to 0.15 mA cm^−2^, implying severe parasitic effect between CGPE and the LMAs. The higher critical current density mainly benefits from the high Li^+^ transfer number of the SGPE in Fig. 1e, which allows for the safer operation of Li metal anode [11].

Further investigation on the reversibility of Li plating/stripping processes on LMAs is performed in Li||Li symmetrical cells (Fig. 3h). Surprisingly, the Li|SGPE|Li symmetric cell delivers excellent interface stability under 0.1 mA cm^−2^ with a smooth voltage profile lower than 0.1 V and an operating time of more than 1000 h. On the contrary, Li|CGPE|Li and Li|LE|Li symmetric cells fail less than 500 h, demonstrating the unstable interface between CGPE/LE and Li anode. Notably, the voltage curves of the Li|CGPE|Li shows a much higher polarization (> 200 mV), which mainly comes from the severe parasitic reaction as discussed later in this article. Typically, when electrolyte contains more free TEP molecules, continuous reduction of TEP by the freshly deposited Li metal occurs, resulting in a thick and unstable passivation SEI layer on LMAs [33]. An ineffective SEI layer limits access to the electrodeposited Li metal, followed by dendritic Li deposition and low Coulombic efficiencies [5].

The same conclusion is also obtained by the impendence of Li symmetric cells in Fig. S10, where the observed semicircle size represents the interfacial resistance (including the contributions from charge transfer and solid electrolyte interphase SEI) [50]. Li|SGPE|Li symmetric cell delivers much smaller interfacial impendence increase than Li|CGPE|Li symmetric cell after 100 cycles, indicating a more stable passivation layer during cycling. Moreover, Li symmetric cell with 1 M-35TFMA GPE electrolyte fails even less than 50 cycles under the same current density of 0.1 mA cm^−2^ during the galvanostatic charge–discharge process (Fig. S11), indicating that the application of dual lithium salt provides a more effective interface protective layer and inhibits the electrolyte from continuous reaction with LMAs compared with a single lithium salt.

As depicted in Fig. S12, the SGPE precursor injected into the cell can fully infiltrate the cathode material during the cell assembly process. From SEM and corresponding energy-dispersive spectroscopy (EDS) of the electrolyte/cathode cross-sectional images taken from a full cell with LFP cathode, S from TFSI^–^ distributes uniformly in the both electrolyte and cathode, which indicates GPEs can penetrate well into cathode, greatly reducing the interfacial impedance.

### Quasi-Solid-State Full-Cell Performance with GPEs

**Fig. 4:**
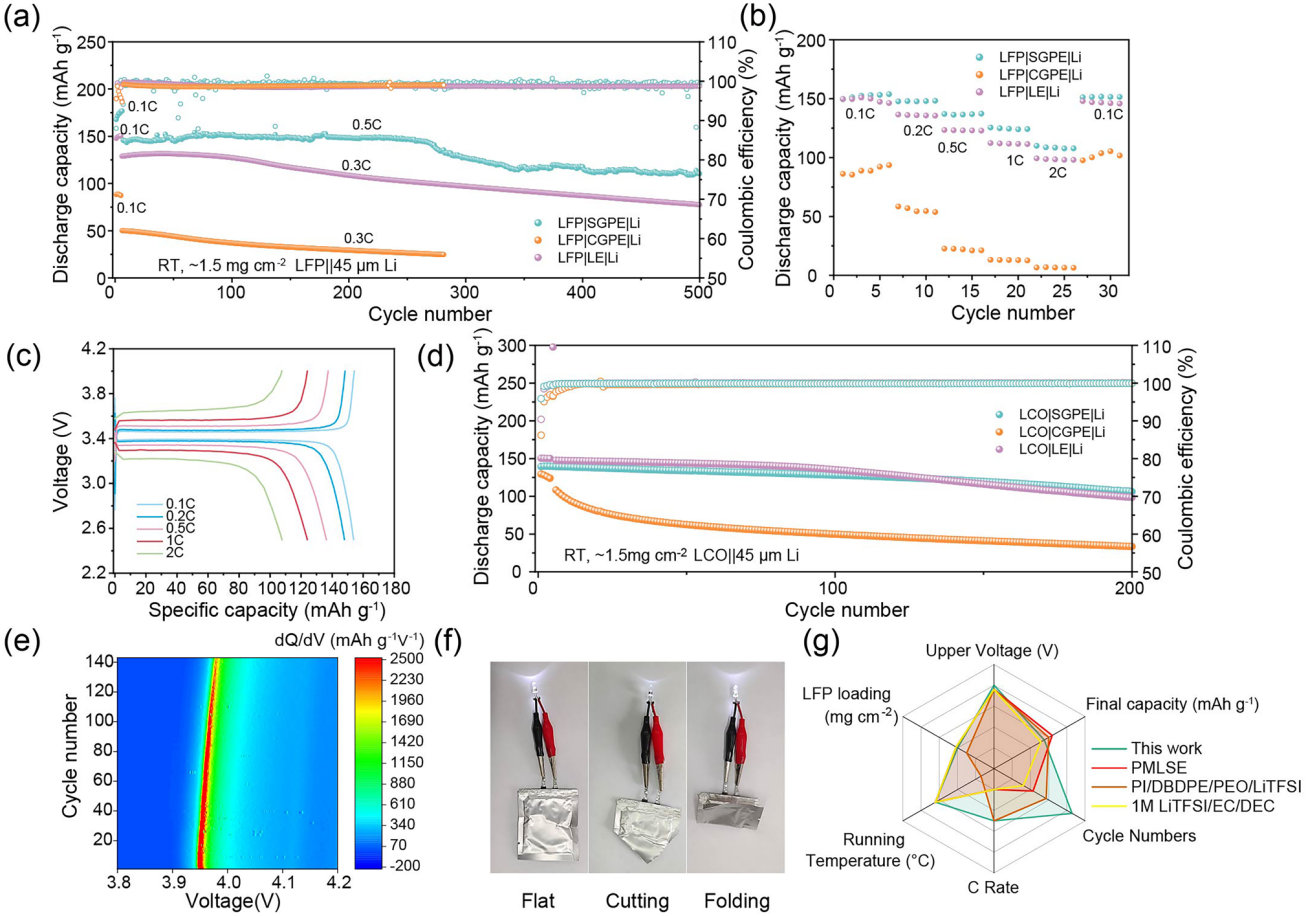
**a** Cycling performances of LiFePO_4_|SGPE|Li cell, LiFePO_4_|CGPE|Li cell and LiFePO_4_|LE|Li cell for 500 cycles after being activated at 0.1 C at room temperature. **b** Rate performance of the LiFePO_4_|SGPE|Li, LiFePO_4_|CGPE|Li and LiFePO_4_|LE|Li full cell at room temperature. **c** Typical charge/discharge curves of LiFePO_4_|SGPE|Li cells under the varied rate from 0.1 to 2 C. **d** Long-term cycling performance of the LiCoO_2_|SGPE|Li cell, LiCoO_2_|CGPE|Li cell and LiCoO_2_|LE|Li cell at 0.2 C after being activated at 0.1 C at room temperature. **e** Contour plot of dQ/dV results of the LiCoO_2_|SGPE|Li cell from the 10th cycle to the 150th cycle. **f** An LED powered by a fully charged LiCoO_2_|SGPE|Li pouch cell after folding and cutting tests. **g** Comparison in LiFePO_4_||Li full cells performance (upper voltage, final capacity, cycle numbers, C rate, running temperature and LFP loading) with the SGEP electrolyte and other reported electrolytes

The long-term cycle performance of cells with GPEs and LE at 0.5 or 0.3 C in 2.5–4.0 V is presented in Fig. 4a. To establish a stable interface, the first few cycles were carried out at 0.1 C. Remarkably, compared with CGPE and LE electrolyte, SGPE enables the system using TEP organic liquid electrolytes with significantly outperformed cycle stability, achieving ∼87.5% capacity retention with an average Columbic efficiency of 98.6% over 250 cycles at 0.5 C. Moreover, although SGPE battery system suffers from slight capacity attenuation after 300 cycles, the stable electrode–electrolyte interface still affords 65.8% capacity retention and an average Columbic efficiency of 98.8% even for 500 cycles. In sharp contrast, the LFP||Li battery with LE exhibits a low capacity (< 130 mAh g^−1^ for the first few cycles at 0.3 C) and fast capacity fading (~ 52.4% capacity retention) after 500 cycles. As for the LFP|CGPE|Li battery, it delivers an ultralow practical capacity of even ~ 50 mAh g^−1^ at 0.3 C. Note that both LFP|CGPE|Li cell and LFP|LE|Li cell show a significant capacity decline at the early cycles, with LFP|CGPE|Li even dropping to ~ 26 mAh g^−1^ after 250 cycles. For LFP|SGPE|Li cell, the capacity fading occurs about 260 cycles with much stable cycle performance. The capacity fading is generally caused by interface deterioration. Therefore, the rapid capacity decline of LFP|CGPE|Li and LFP|LE|Li cells may be caused by uncontrolled and continuous electrolyte depletion, leading to unfavorable side reactions and slow interfacial ion transport on both electrodes. On the contrary, a delayed capacity attenuation for LFP|SGPE|Li cell reasonably indicates side reaction of TEP molecules has been depressed with the design of SGPE system, for which the protective mechanism will be further discussed subsequently. Further charge/discharge details are presented in Fig. S13. Batteries with SGPE and LE exhibit similar charge/discharge voltage at ~ 3.4 V, which corresponds to the phase transition between LiFePO_4_ and FePO_4_ [39]. The cycle performance demonstrated by LFP|SGPE|Li cell is more favorable, with a reversible specific capacity of 110.4 mAh g^−1^ delivered at 0.5 C after 500 cycles, which is even better than that of LE and CGPE at lower current density of 0.3 C. Long-term cycle performances of LiFePO_4_||SGPE||Li cell at 1 and 2 C were also performed and the results are shown in Fig. S14. It is obvious that the capacity decreases under the current density of 1 or 2 C, with 96.1 mAh g^−1^ for 1 and 72.1 mAh g^−1^ for 2 C after 500 cycles. Therefore, for a comprehensive and balanced consideration, current density of 0.5C is more appropriate for LiFePO_4_||SGPE||Li cell to deliver long-term cycle.

Rate performance of batteries from 0.1 to 2 C and corresponding charge–discharge profiles are presented in Fig. 4b. Obviously, the rate performance of LFP|SGPE|Li is much better than that of LFP|CGPE|Li and LFP|LE|Li, which may originate from the low ionic conductivity and nomadic phosphates of CGPE on the electrode–electrolyte interface. The cell with SGPE shows favorable rate performance, delivering capacity of 153.8, 147.9, 136.3, 124 and 107.7 mAh g^−1^ at 0.1, 0.2, 0.5, 1 and 2 C, respectively, while those of CGPE are 88.8, 54.6, 21.3, 12.6 and 6.6 mAh g^−1^ and LE are 146.26, 135.54, 122.92, 111.46 and 98.06 mAh g^−1^. Corresponding charge/discharge voltage curves of cells with SGPE and CGPE are presented in Figs. 4c and S15, respectively, where specific capacity of SGPE cell is higher than CGPE and LE cell as expected, validating the good rate performance of the cell with SGPE under different rates between 2.5 and 4.0 V.

Additionally, LiCoO_2_ (LCO)||Li cells were also assembled to evaluate the compatibility of GPEs with the high-voltage cathode at the cutoff voltage of 4.2 V. As shown in Fig. 4d, LiCoO_2_|SGPE|Li cell delivers a high initial discharge capacity of 139.4 mAh g^−1^ and a specific capacity of 106.1 mAh g^−1^ after 200 cycles, which retains 76.1% of the initial capacity with a high average columbic efficiency of 99.9%, significantly higher than the cells with CGPE. Detailed charge–discharge profiles of the 4.2 V LCO cells with GPEs and LE electrolyte are illustrated in Fig. S16, and the comparative results validate the interfacial compatibility between SGPE and the high-voltage LCO cathode, which is beneficial to the application of high-energy-density system. To further determine the change of cycle performance and kinetic process during cycling, derivative of capacity versus voltage (dQ/dV) was applied to analyze the change of oxidation peaks. As shown in Figs. 4e and S17, the contour plot of dQ/dV results indicates that the LCO|SGPE|Li cell presents a significantly smaller potential shifts and capacity attenuation compared to the LCO|CGPE|Li cell for the 10–150 cycles, which demonstrates stable interfaces and improved kinetic process [51]. Subsequently, pouch cells were assembled to demonstrate the safety and flexibility of LFP|SGPE|Li battery. As is shown in Fig. 4f, the assembled pouch cell could consistently illuminate white light emitting diodes (LEDs) when experiencing destructive bending, cutting and penetration tests, verifying its excellent safety and flexibility. Such battery performances under harsh conditions far exceed the normal safety requirements. Figure 4g compares the electrochemical performance of SGPE with other modified electrolytes reported for LFP||Li cells and more comparisons are shown in Table S2. In contrast to 1 M LiTFSI/EC/DEC liquid electrolyte, PMLSE gel electrolyte and PI/DBDPE/PEO/LiTFSI polymer electrolytes, SGPE has more balanced performance in terms of upper voltage, final capacity, cycle numbers, C rate, running temperature and LFP loading, which is expected to be a promising electrolyte for LMBs.

### SEI/CEI Characterization and Growth Model in Multiscale Design Principle

**Fig. 5:**
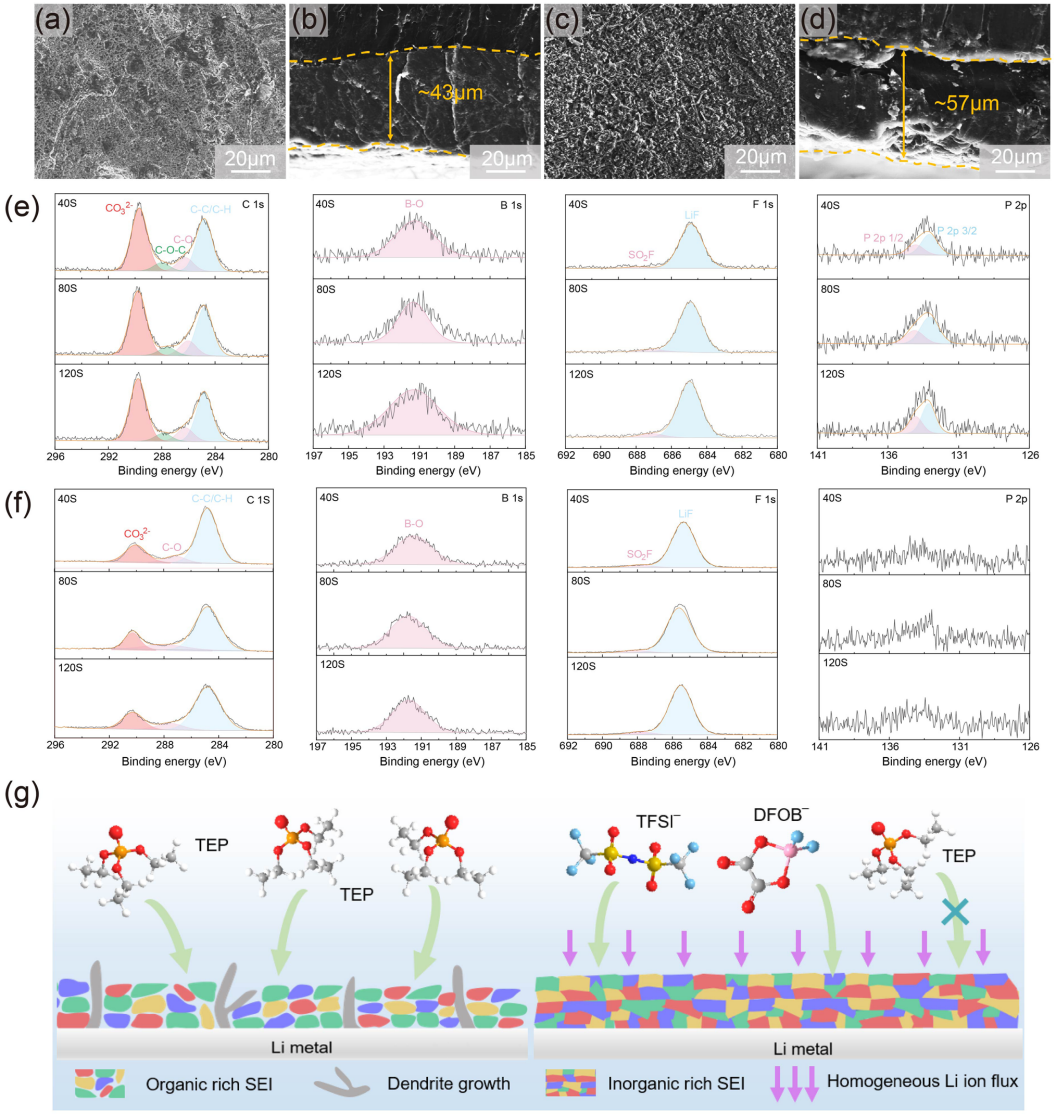
Morphologies and components analysis of lithium metal anodes. Top views of SEM images of deposited Li with **a** SGPE and **c** CGPE after 200 cycles at the current density of 0.1 mA cm^−2^ and a capacity of 0.05 mAh cm^−2^ in Li||Li cells. Cross-sectional views of SEM images of Li plating morphologies in the **b** SGPE and **d** CGPE after 200 cycles at the current density of 0.1 mA cm^−2^ and a capacity of 0.05 mAh cm^−2^. XPS depth profiles of C 1*s*, B 1*s*, F 1*s*, P 2*p* in the Li metal anode after 40 cycles for **e** CGPE and **f** SGPE. **g** Schematic representation of the solid electrolyte interface (SEI) formed on the Li metal electrode with SGPE and CGPE, respectively

Above all, TEP-contained SGPE delivers excellent cycle performance. However, it is noted that TEP is reported to easily react with Li metal and result in dramatic battery failure [33]; therefore, we speculate that SGPE may incline to form a stable protective layer on the surface of Li metal benefiting from the suppressed parasitic side reactions between TEP and Li metal. SEM and XPS measurements were taken to study the morphologies and components of the SEI layer on the electrodes. Typical top-view morphologies of deposited Li on Li anode after 200 cycles were characterized by SEM in Fig. 5a, c. A loose layer with distinguished dendritic Li is observed on the surface with CGPE. In contrast, SGPE leads to a smooth and dense surface, which shows a more controlled deposition process. The cross-sectional view in Fig. 5b, d indicates that thickness of the passivation layer (dead Li) is ~ 43 μm for SGPE, less than ~ 57 μm for CGPE. Such Li-deposited morphology explains the cycling life of symmetric Li||Li cells in Fig. 3h. The raw SEM images are further shown in Fig. S18.

Variations of SEI species on the cycled Li anode with SGPE and CGPE were discussed by in-depth XPS. For CGPE sample, high-resolution C 1*s* includes C-C/C-H (~ 284.8 eV), C-O (~ 286.3 eV), C-O-C (~ 288.1 eV) and CO_3_^2−^ (~ 289.6 eV) [52] signals (Fig. 5e), which may come from the decomposition of TEP, HEMA polymer matrix and Li salt anions of CGPE. Intensive CO_3_^2−^ peaks correspond to severe decomposition of electrolytes [39], which causes a rapid increasing internal resistance, and eventually leads to battery failure. The strong signals of LiF (~ 687 eV) and B-O (~ 191.8 eV) exist in the whole etching process, demonstrating sufficient decomposition of Li salts in all cycles, which is in agreement with the absence of C-F and B-F signals in C 1*s* and B 1*s* spectra. Peaks at ~ 133 eV in the P 2*p* spectra correspond to the existence of polyphosphates resulting from the decomposition of TEP. The content of P species remains invariable with etching depth increases, which implies continuous decomposition of TEP solvents. Therefore, the SEI on the Li anode surface with CGPE is primarily composed of organic decomposition species. The extensive participation of TEP solvent in SEI formation will obviously raise the organic contents of SEI, leading to thick, soft but weak protection layer.

XPS of SGPE system in Fig. 5f demonstrates many differences. In C 1*s* spectra, the C-C and C-O-C signals can be observed at almost same ratio and stay invariable during the etching process. Interestingly, its peak intensity of CO_3_^2−^ shows obvious decline compared to CGPE system, suggesting a great inhibiting effect for decomposition of phosphate solvent and polymer matrix. The completely decomposed product of TFSI^−^/DFOB^−^ anions (LiF and B-O species) can also be observed when cycled with SGPE, revealing the domination of LiF/B-O in F/B-containing species of SEI and preferential-decomposed tendency to form the protective passivation layer [6]. What is more, in sharp contrast to CGPE system, which has intensive P signals, there are very weak peaks in P 2*p* spectra of SGPE system, indicating less TEP solvents experience parasitic reaction and participate in the formation of SEI on Li metal anode, which is significant to produce an inorganic-rich SEI. With sputtering depth, the intensity of C, LiF, B-O and P peaks in the SGPE system almost keep unchanged, indicating that SEI layer rich in inorganic species is uniform over the anode.

SEI layer plays important roles to buffer the volume change of Li and induce uniform Li^+^ flux through interface during the plating/stripping process. The more stable Li deposition with SGPE should come from the more stable SEI. Figure 5g depicts the structure illustration of the organic-rich SEI in CGPE and the inorganic-rich SEI in SGPE according to XPS analysis. Organic-rich SEI is proposed to improve the mechanical flexibility, but its strong lithiophilicity also enable SEI with the same volume change as Li during recurrent Li plating/stripping. Therefore, the cracking of the organic-rich SEI is unavoidable [53]. The fissures accelerate the formation of locally concentrated Li-ion flux, which leads to an uneven Li deposition with dendritic morphology that penetrates the separator, thus causing continuous electrode/electrolyte depletion and short circuit [54]. What is more, the organic components deliver low lithium ionic conductivity, which aggravates an unstable Li/electrolyte interface. Therefore, the much higher interfacial impendence of Li|CGPE|Li cell than Li|SGPE|Li (Fig. S10) can be elucidated by the unfavorable SEI layer, which delivers inefficient Li^+^ diffusion through the interface. In contrast, a much stable SEI layer forms for SGPE (Fig. 5g), containing high proportion of LiF, B-O inorganic species, which shows lithiophobicity with a high interfacial energy with Li metal. The SEI with such a uniform structure is stable, where inorganic LiF and B-O assure sufficient Li^+^ conductivity and boost the Li lateral diffusion over SEI/Li interface [53]. Meanwhile, the organic species endure the large volume expansion of Li anode during cycling, thus resulting in highly reversible Li stripping/plating and a low pulverization [6].

**Fig. 6:**
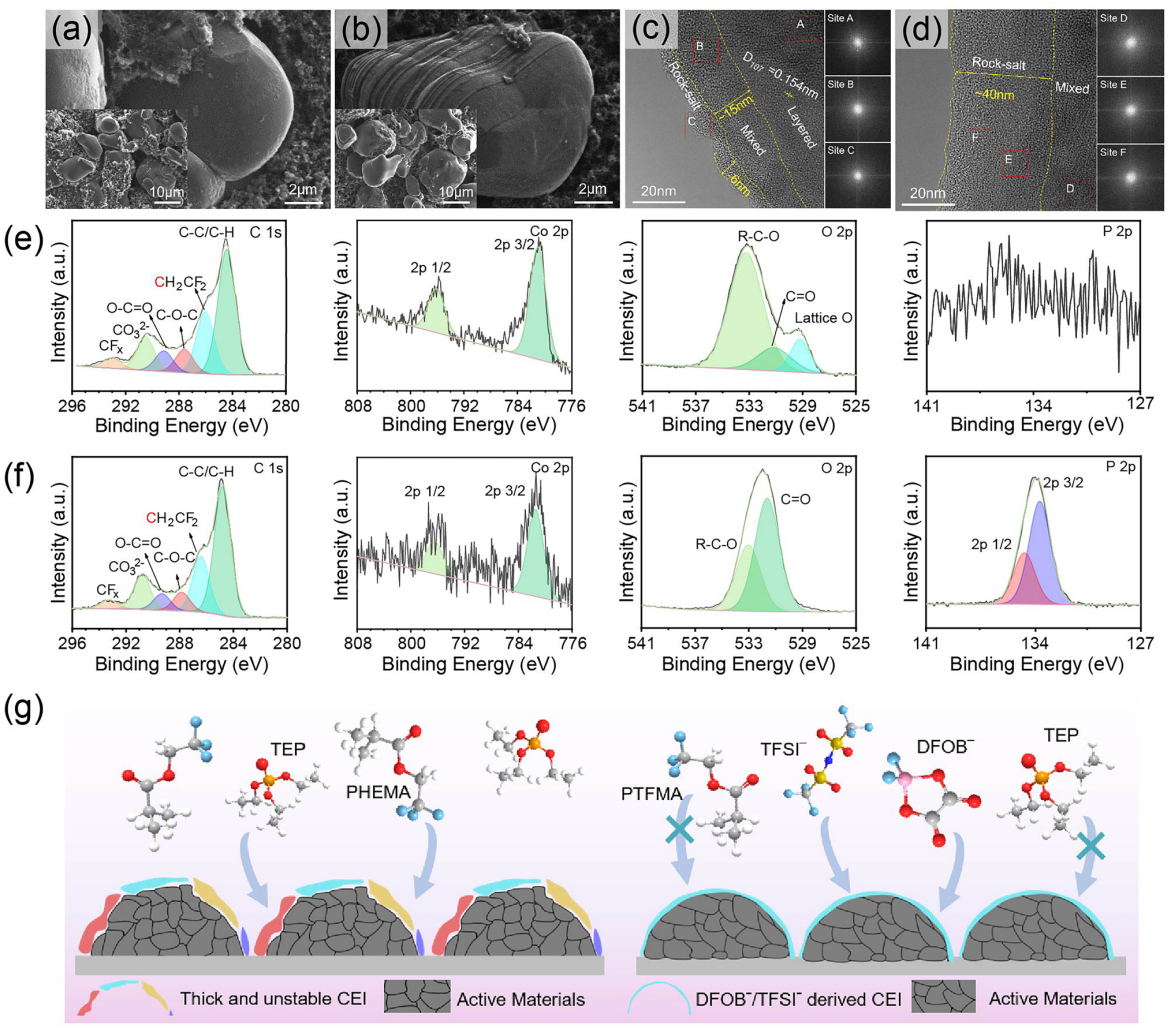
Characterizations of the cycled LiCoO_2_ cathodes and CEIs with SGPE and CGPE. SEM images of LiCoO_2_ cathodes cycled in **a** SGPE and **b** CGPE. High-resolution TEM and FFT images of LiCoO_2_ particles cycled in **c** SGPE and **d** CGPE. XPS spectra for C 1*s*, Co 2*p*, O 2*p* and P 2*p* of the LiCoO_2_ cathodes cycled in **e** SGPE and **f** CGPE after 30 cycles. **g** Schematic illustration of uniform and damaged cathode electrolyte interface (CEI) formed on the LiCoO_2_ cathode with SGPE and CGPE, respectively

The interface stability between GPE and high-voltage cathode is critical for the application of high-energy-density batteries. Therefore, analysis on cycled 4.2 V LCO cathodes/GPEs interface was also performed to reveal the reasons for the excellent stability of SGPE over CGPE. The cathodes investigated upon different measurements were disassembled from 4.2 V LCO||Li batteries after 30 cycles. As shown in Fig. 6a, b, there are broken strips on the surfaces of LCO particles working with CGPE, while that with SGPE has a relatively smooth surface and keep intact after cycling. Surface structure of cycled LCO were further explored by high-resolution transmission electron microscopy (HRTEM). As shown in Fig. 6c, d, the nanostructures confirmed by FFT patterns show that phase transformation occurs from surface to bulk during cycling, which consists of rock salt phase (Site C, E, F), mixed phase (Site B) and layered structure (Site A). For LCO with SGPE, the surface undergoes a slight phase transition, with only about 6-nm-thick disordered rock salt phase and a large part of layered structure in the bulk. In contrast, the cathode with CGPE undergoes severe phase transition, with ~ 40 nm disordered rock salt phase and a thick mixed layer of ~ 20 nm. There is almost no layered structure preserved, suggesting a visible structural collapse of LCO. Moreover, as shown in Fig. S19, XRD patterns present that there is no detectable difference in bulk structure between pristine and cycled LCO in SGPE, which is in sharp contrast to the patterns in CGPE. Such results indicate that the detrimental phase transition is effectively suppressed in SGPE. Generally, the preservation of integrated structure in SGPE can be attributed to the formation of a uniform and thin CEIs [33, 52], which demonstrates less parasite reaction between SGPE and LCO particles.

XPS was used to further characterize the chemical composition of CEIs on the LCO cathodes (Fig. 6e, f). In the C 1*s* spectra, although the signals correspond to PVDF binder and super P, the one cycled with SGPE has a weaker C signal compared to that with CGPE, indicating fewer organic decomposition species. For the Co 2*p* analysis, signals of Co^2+^ from LCO with CGPE show a relatively sparse peak intensity than the one with SGPE during cycling [55], indicating a much thicker in situ formed passivation layer over LCO particles in CGPE electrolyte [52]. The O 1*s* XPS analysis results show that the lattice oxygen (~ 529.3 eV) peak of LCO at the SGPE/LCO interface is stronger, which also confirms that the CEI layer was much thinner in SGPE [7]. Significantly, there are barely P signals observed in the battery with SGPE. It could be inferred that the proportion of free TEP is reduced in SGPE, thus suppressing its attack to the cathode [6]. Meanwhile, more inorganic signals of B-O (190.2 eV) and LiF (684.8 eV) can be observed in SGPE system (Fig. S20), which is mainly due to the decomposition of lithium salt anions [33].

Therefore, combining the results of SEM, TEM, XRD and XPS, we conclude that an effective passivation CEI layer (inorganic-rich) forms on the surface of LCO cathode with SGPE. However, in sharp contrast, the CEI formed on LCO surface is organic-rich in CGPE system. The schematic representation is depicted in Fig. 6g. Generally, in CGPE, some solvents and polymer matrix decompose into organic species under high voltage, which triggers the generation of thick and unstable CEI. In comparison, an inorganic-rich CEI is established in the SGPE and it is believed to maintain the structure integration, suppress the dissolution of TMs and promote effective Li^+^ transport, thereby assuring more remarkable cycling stability under high voltage [55]. Such CEI structure explains the cycling life and capacity retention for the LCO||Li cells with different GPEs in Fig. 4f.

### Revealing Molecular Mechanism for Multiscale Design

**Fig. 7:**
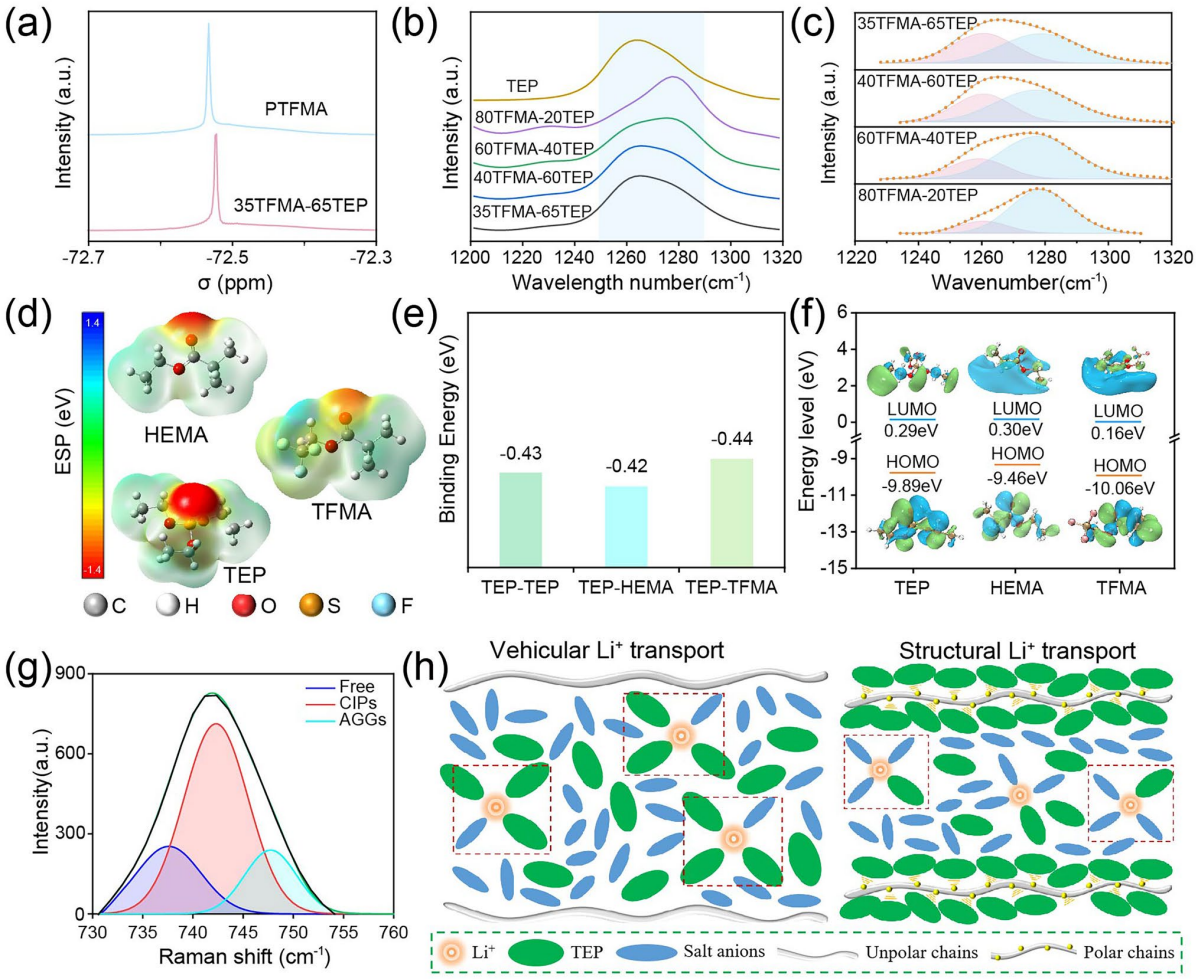
**a** ^19^F-NMR spectra of PTFMA with and without TEP solvents. **b** FTIR spectra of -P = O peaks in TEP-based samples with different TFMA concentrations (more details are depicted in the Experimental Section). **c** Fitted results of FT-IR curves about the status of TEP species (free TEP and anchored TEP). **d** The optimized geometric configurations and electrostatic potential of TEP, TFMA and HEMA. **e** The binding energy of TFMA-TEP, HEMA-TEP and TEP-TEP. **f** HOMO and LUMO energy levels for TFMA, HEMA, TEP. **g** Deconvolution of peaks of the S-N-S stretching vibrational mode (CIP: contact ion pair, AGG: aggregated ion pair) in SGPE electrolyte. **h** Schematic of the interaction between polymer chains and TEP molecules in SGPE electrolyte compared to the CGPE electrolyte

Obviously, SGPE-based batteries exhibit excellent interfacial compatibility with both electrodes by constructing an effective passivation layer. Although TEP is considered to be unstable during cycling, especially on the LMAs, its attack to both sides of electrodes are almost negligible in SGPE-based batteries. Therefore, we reasonably speculate that TFMA polymer matrix in SGPE limits the free movement of TEP molecules through intermolecular interactions, thus suppressing the parasitic side reactions between TEP and electrodes. To reveal the interaction between TFMA and TEP molecules, different samples including pure TEP, TFMA, HEMA, TFMA-TEP and HEMA-TEP were systematically characterized by nuclear magnetic resonance (NMR) and FTIR spectroscopy. As shown in the ^19^F NMR spectra in Fig. 7a, the peak from -CF_3_ belonging to PTFMA in 35TFMA-65TEP exhibits more significant downfield displacement than PTFMA, demonstrating that the electron cloud density exchange occurs on F atoms [31], which belongs to a dipole–dipole interaction between TFMA chains and TEP solvents. The intermolecular interaction was also verified by FTIR spectroscopy (Figs. 7b and S21). For TFMA-TEP system (Fig. 7b), the -P = O peak of TEP at ~ 1260 cm^–1^ delivers obvious blue shift as the content of TFMA increases. In contrast, the peaks barely show any shifts in HEMA-TEP system (Fig. S21), even the content of HEMA increases. Generally, an increase in wavenumbers can be ascribed to the change of bond length, which is susceptible to the interaction between functional groups [56]. The results indicate the presence of dipole–dipole interaction between TFMA and TEP molecules. Therefore, a logical anchoring effect is proposed between TEP molecules and PTFMA matrix, which reduces free TEP and dramatically improves the electrochemical compatibility of TEP molecules. To further reveal the TEP state, curves in Fig. 7b were further analyzed in the regions of 1225–1320 cm^−1^ and the fitted results are shown in Fig. 7c, where the signals at ~ 1260– ~ 1278 cm^–1^ are indexed to free TEP and anchored TEP, respectively. As TFMA increases, anchored TEP increased from 55.6% to 81.6%, indicating the well control to TEP state. Notably, the 35TFMA-65TEP sample used in SGPE dedicates coordinated TEP up to 55.6%, which is not very high but effective for better stability as shown before. Of course, it does not mean the higher TFMA the better, because high TFMA contents leads to low ionic conductivity. Proper reducing the ratio of free TEP by introduction of polar -CF_3_ group on the polymer chains can effectively enhance the compatibility with both electrodes.

Theoretical calculations were also used to further investigate the contrastive internal interactions of SGPE and CGPE. As shown in Fig. 7d, the electrostatic potential (ESP) was first calculated to figure out the charge distribution of TEP, HEMA and TFMA, which significantly influences the interaction between solvents and polymer network. Obviously, the negative charge almost focuses on O atoms of -P = O groups in TEP molecules, which is conducive to coordinate with electropositive polymer network. Moreover, due to the much higher electron-withdrawing effect of -CF_3_ element than -CH_3_ group [38], PTFMA matrix presents more polarized characteristic of the -CH_2_ groups, thus augmenting the coordination capability of TFMA with electronegative P = O group in TEP molecules. Binding energy of geometric configuration optimized systems was also calculated to compare the molecular interaction (Fig. 7e). TFMA-TEP exhibits the biggest binding energy of −0.44 eV compared with HEMA-TEP (−0.42 eV) and TEP-TEP (−0.43 eV). It results in much stronger TEP-TFMA interactions than TEP-TEP interactions in SGPE system, while TEP in CGPEs prefers TEP-TEP dipole–dipole interactions with itself rather than with HEMA. The large binding energy would anchor TEP molecular on polymer matrix such as in SGPE, effectively suppressing the activity of TEP on electrodes.

As proved before, anchoring effect of solvents in SGPE can effectively enhance the redox stability of TEP molecules, where more stable SEI/CEI can be constructed by suppressed decomposition of TEP (Figs. 5g and 6g). To further validate the influence of polymer skeleton on the redox stability in GPE system, molecular orbital energy levels were carried out (Fig. 7f). The redox stability is tightly associated with the lowest unoccupied molecular orbital (LUMO) and highest occupied molecular orbital (HOMO) levels. The low LUMO level means to be readily reduced on anode, while high HOMO level is easily to be oxidized on cathode. As shown in Fig. 7f, the LUMO are calculated to be LUMO_HEMA_ (0.30 eV) ≈ LUMO_TEP_ (0.29 eV) > LUMO_TFMA_ (0.16 eV). Therefore, for CGPE, besides decomposition of TEP solvents on lithium anode, PHEMA matrix also decomposes almost at the same time and results in high content of P, C-rich organic components in the SEI. However, for SGEP, the LUMO becomes much lower than that of TEP, which means PTFMA matrix can be reduced earlier than TEP, mitigating decomposition of TEP and increasing the content of LiF content in SEI layer [39, 57], which contributes to a more uniform Li ion flux through the interface. For the HOMO levels, it delivers HOMO_HEMA_ (−9.46 eV) > HOMO_TEP_ (− 9.89 eV) > HOMO_TFMA_ (− 10.06 eV). As a result, PHEMA delivers an earlier decomposition than TEP, leading to organic-rich CEI layer in CGPE system, which results in uneven Li ion transport and deteriorated cycling stability. Admiringly, the PTFMA delivers the much lower HOMO level compared to TEP in the SGPE, suggesting the extraordinary oxidation stability by the introduction of -CF_3_ group. Therefore, despite functions as polar chains to anchor TEP solvents, the PTFMA skeleton also delivers intrinsic improved stability according to the molecular orbital energy level theory.

Besides, the Raman results also verified the coordination structure between PTFMA polymer chain and TEP molecules. In the range from 740 to 750 cm^−1^ in Figs. 6g and S22, peaks have three different dissociation states of TFSI^−^ anions: free TFSI ions, contact ion pairs (CIPs, TFSI anions interacting with a single Li ion) and aggregated ion pairs (AGGs, TFSI anions interacting with two or more Li ions) [58]. As demonstrated in the peak deconvolution results, TFSI ions interact with more Li ions in 1 M-SGPE compared with the 1 M-CGPE, which could be ascribed to the fact that less percent of free TEP participating in the solvation process of Li^+^ in 1 M-SGPE than 1 M-CGPE, forming a more concentrated coordination structure. The anions coordinated structure may benefits from the fact that more TEP molecules are in the inactive anchored status, and therefore, their capacity to solvate Li^+^ has been constrained [59]. The anions coordinated structure strongly implies that direct contacting between TEP and electrodes has been reduced and anions are more readily participating into the formation of passivation layer, which increases the content of inorganic species in SEIs/CEIs and improves the electrochemical performances.

Briefly, the interactions between TFMA and TEP are shown in Fig. 7h, in which the lone pair electrons on the oxygen of -P = O in TEP molecules are strongly attracted to the electropositive -CH_2_ atoms in the PTFMA polymer, leading to a completely uniform super-molecular structure rather than simply mixing with each other in CGPE [31, 56]. This network with a coordination structure can suppress the volatilization of TEP solvents, effectively attenuate the unfavorable motion of TEP and prevent TEP from directly contacting and reacting with electrodes, thus guaranteeing an outstanding electrochemical stability, thermal stability and interfacial compatibility. With more nomadic TEP anchored to the TFMA chains, the Li^+^ migration mode tends to be regulated from the sluggish vehicular transport to fast structural transport. Therefore, an effective strategy is proposed to design high-performance gel polymer electrolytes by designing supramolecular chemistry network through the targeted introduction of polar TFMA polymer matrix to TEP-based electrolyte, which is helpful to promote the application of high-energy-density lithium metal batteries.

## Conclusions

In conclusion, we develop a nonflammable GPE by introducing flame-retardant TEP electrolyte to the in situ polymerized PTFMA skeleton. With rational formulation of electrolyte ingredients, the well-designed SGPE features outstanding ionic conductivity (1.03 mS cm^−1^ at 30 °C), admirable nonflammability and appropriate mechanical strength. A unique coordination structure between PTFMA network and TEP solvents has been proved by the technique of NMR/FT-IR/Raman spectroscopy and DFT simulation. Therefore, the special intermolecular interaction not only ensures superior electrochemical stability and thermal stability, but also contributes to limited solvents mobility, which significantly weakens Li^+^-solvent interactions, inhibits electrode–electrolyte side reactions and promotes the formation of anion-derived SEIs/CEIs. As a result, the formation of B/F-rich inorganic components in the SEIs/CEIs is proved to simultaneously inhibit lithium dendrite growth, enhance the reversibility of Li metal battery and suppress irreversible phase transition of 4.2 V LiCoO_2_ cathode. Moreover, interface stability of polymer chains themselves is also regulated with the introduction of -CF_3_ group, including the formation of LiF-rich SEI with limited -CF_3_ groups decompose and improved oxidation stability. Consequently, the Li|SGPE|Li cell demonstrates long-term stability for over 1000 h at 0.1 mA cm^−2^ with high stripping/plating reversibility. The LFP|SGPE|Li battery enables an admirable specific capacity, with a capacity retention of ∼65.8% and an average Coulombic efficiency of ~ 98.8% over 500 cycles. The as-assembled 4.2 V LiCoO_2_||Li metal battery also delivers superior cycling stability at room temperature, with capacity retention of 76.1% after 200 cycles. The results highlight the pivotal role of polymer network–solvents interactions and recapitulate the promising electrolyte design for the future development of high-energy-density LMBs.

## Supplementary Information

Below is the link to the electronic supplementary material.

Supplementary file1 (DOCX 4129 KB)
