# Supplementary material for: Molecule-Level Multiscale Design of Nonflammable Gel Polymer Electrolyte to Build Stable SEI/CEI for Lithium Metal Battery
Source: Nanomicro Lett. 2024 Sep 27;17:18. doi: 10.1007/s40820-024-01508-z (PMC11427645; doi:10.1007/s40820-024-01508-z)
Supplement: Supplementary file 1 — Supplementary file1 (DOCX 4129 KB) [file 40820_2024_1508_MOESM1_ESM.docx]

Supporting Information for

**Molecule-Level Multiscale Design of Non-Flammable Gel Polymer Electrolyte to Build Stable SEI/CEI for Lithium Metal Battery**

Qiqi Sun^1^, Zelong Gong^1^, Tao Zhang^1^, Jiafeng Li^1^, Xianli Zhu^1^, Ruixiao Zhu^1^, Lingxu Wang^1^, Leyuan Ma^1^, Xuehui Li^1^, Miaofa Yuan^1^, Zhiwei Zhang^1^, Luyuan Zhang^1^, Zhao Qian^1,*^, Longwei Yin^1,*^, Rajeev Ahuja^2^ and Chengxiang Wang^1,*^

^1^ Key Laboratory for Liquid-Solid Structural Evolution and Processing of Materials (Ministry of Education), School of Materials Science and Engineering, Shandong University, Jinan 250061, P. R. China

^2^ Condensed Matter Theory, Department of Physics and Astronomy, Uppsala University, Uppsala 75120, Sweden

*Corresponding authors. E-mail: [qianzhao@sdu.edu.cn](mailto:qianzhao@sdu.edu.cn) (Zhao Qian); [yinlw@sdu.edu.cn](mailto:yinlw@sdu.edu.cn) (Longwei Yin); [wcxmat@sdu.edu.cn](mailto:wcxmat@sdu.edu.cn) (Chengxiang Wang)

**Supplementary Figures and Tables**


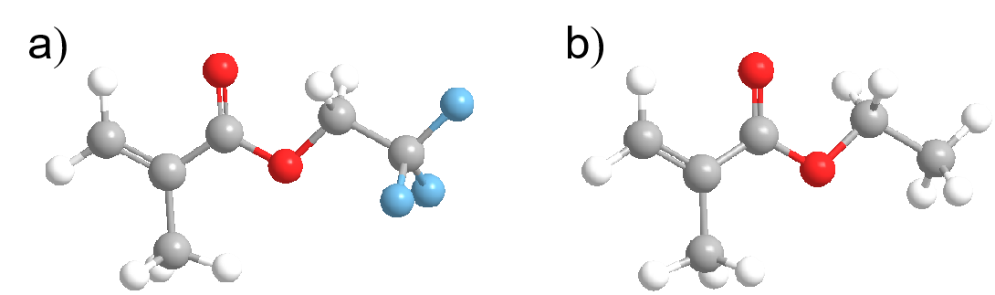


**Fig. S1** Molecular structures of TFMA (**a**) and HEMA (**b**)


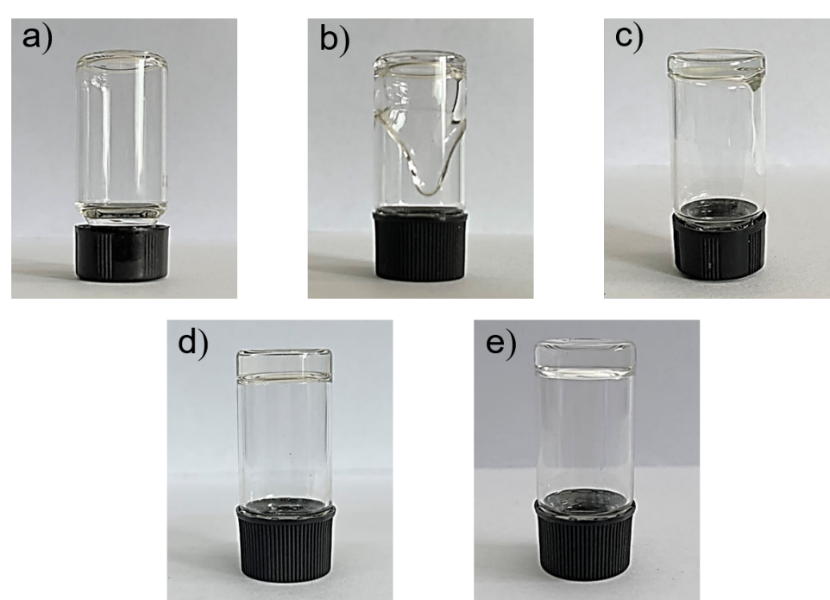


**Fig. S2** Optical photographs of in-situ prepared (**a**) 10TFMA-0.5LF-0.5LB, (**b**) 20TFMA-0.5LF-0.5LB, (**c**) 30TFMA-0.5LF-0.5LB, (**d**) 35TFMA-0.5LF-0.5LB and (**e**) 35HEMA -0.5LF -0.5LB within vials


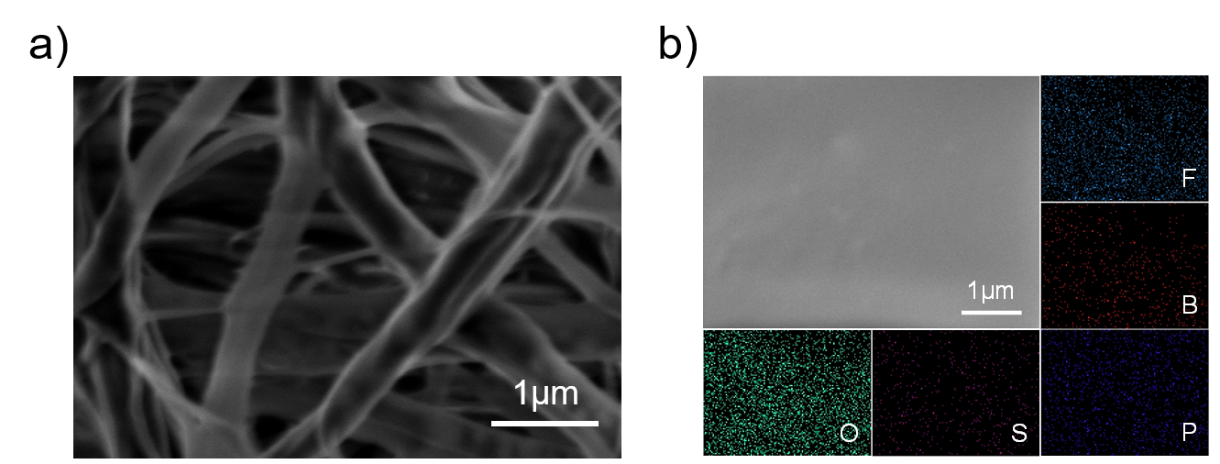


**Fig. S3** SEM images of **a**) original cellulose separator and **b**) obtained GPE with corresponding element mapping


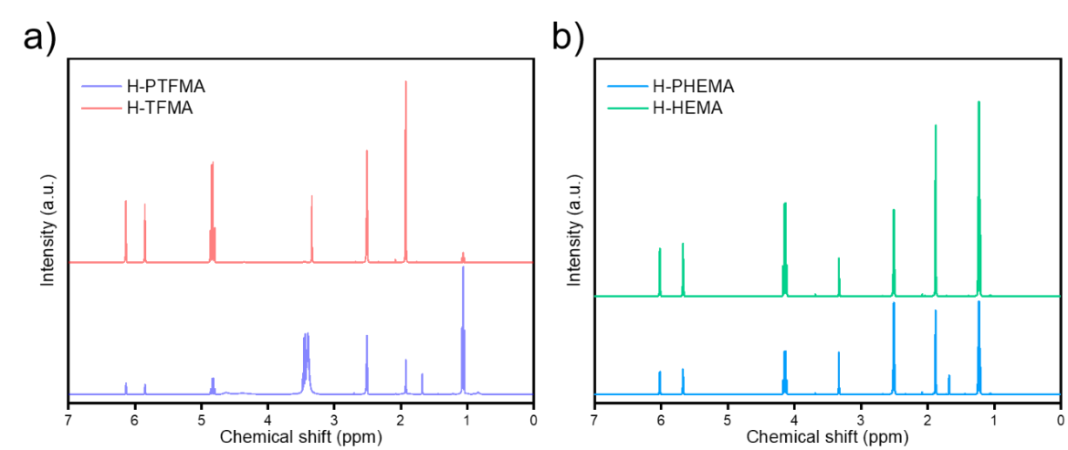


**Fig. S4** ^1^H nuclear magnetic resonance (^1^H NMR) spectra of **a**) TFMA and **b**) HEMA monomer before and after polymerization


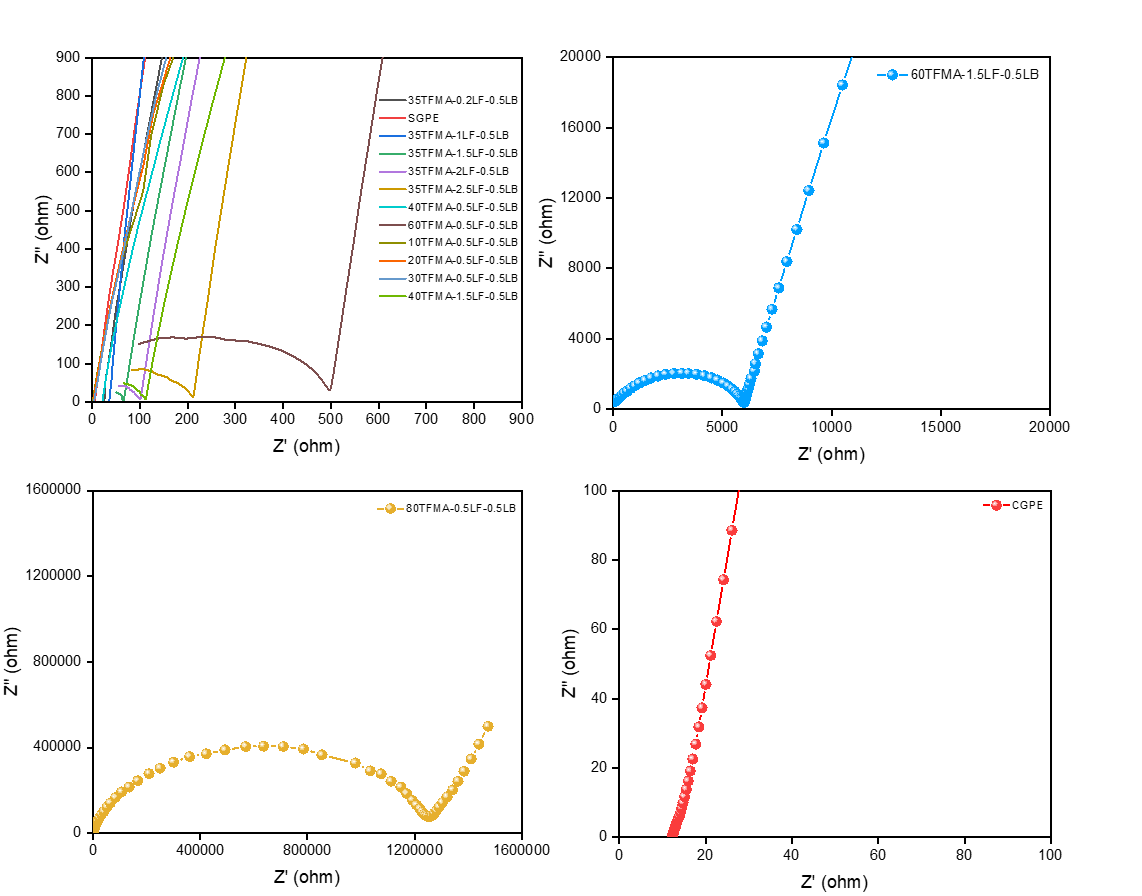


**Fig. S5** Nyquist plots of various electrolytes obtained with SS|GPEs|SS at room temperatures


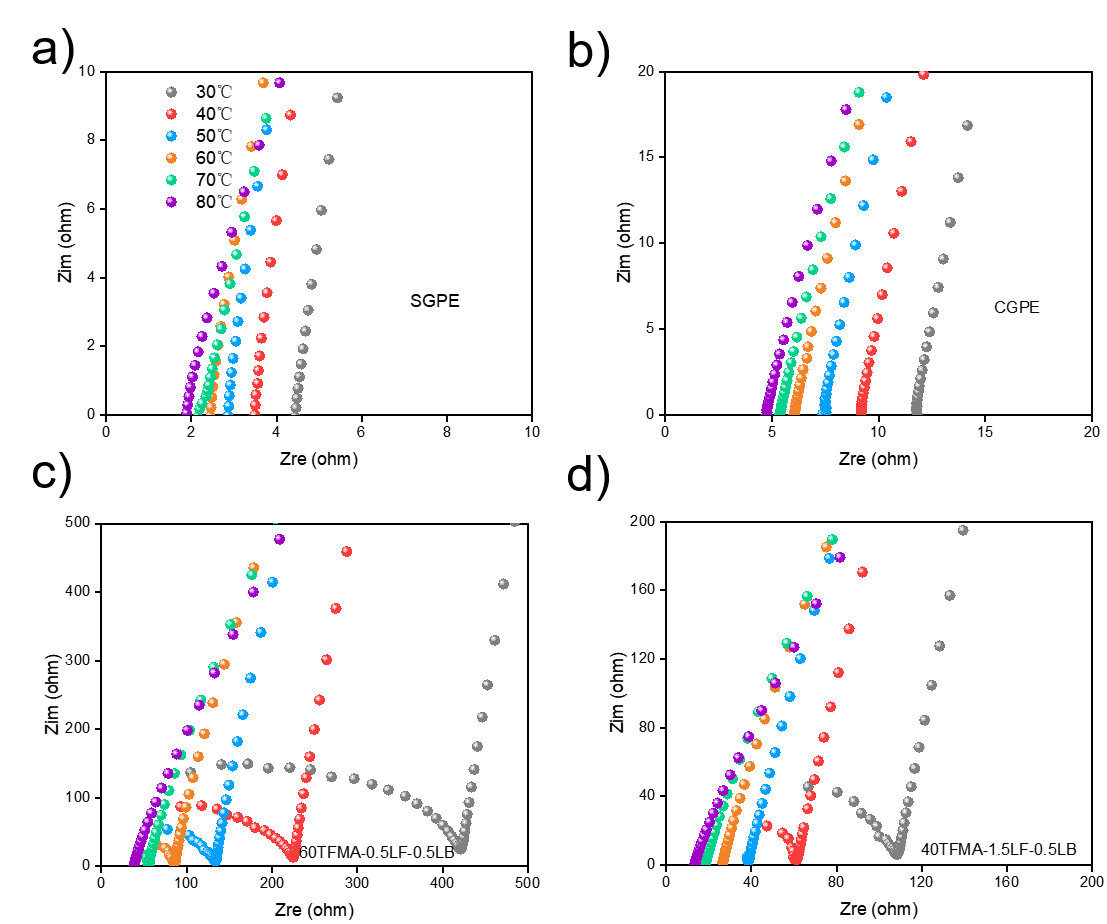


**Fig. S6** Nyquist plots of **a**) SGPE electrolyte, **b**) CGPE electrolyte, **c**) 60TFMA-0.5LF-0.5LB electrolyte and **d**) 40TFMA-1.5LF-0.5LB electrolyte under various temperature

**Fig. S7** Chronoamperometry profile collected from a symmetric Li|CGPE|Li cell (The inset corresponds to the EIS plots before and after chronoamperometry)


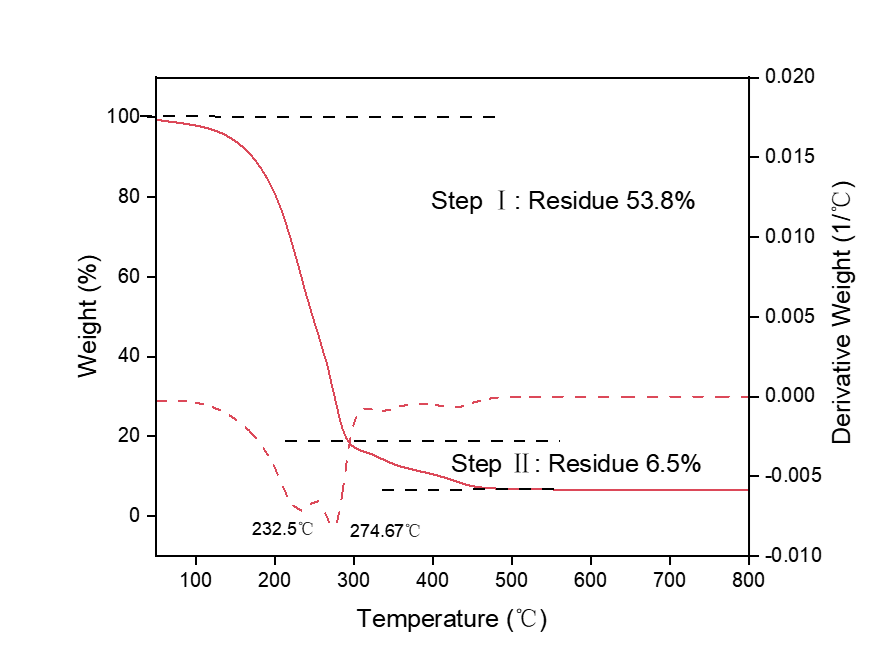


**Fig. S8** TGA thermograms of the CGPE electrolyte

**Fig. S9** Stress-strain curves of GPEs


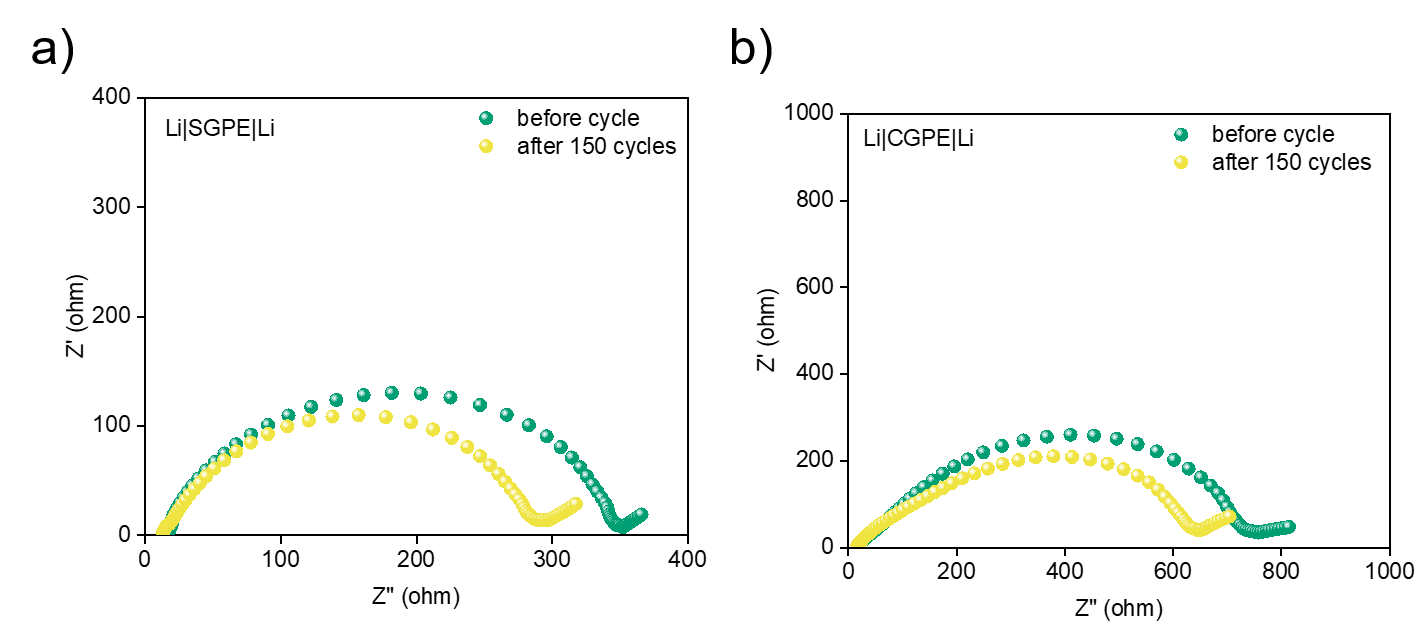


**Fig. S10** EIS spectra of **a**) Li|SGPE|Li and **b**) Li|CGPE|Li cells after 150 cycles


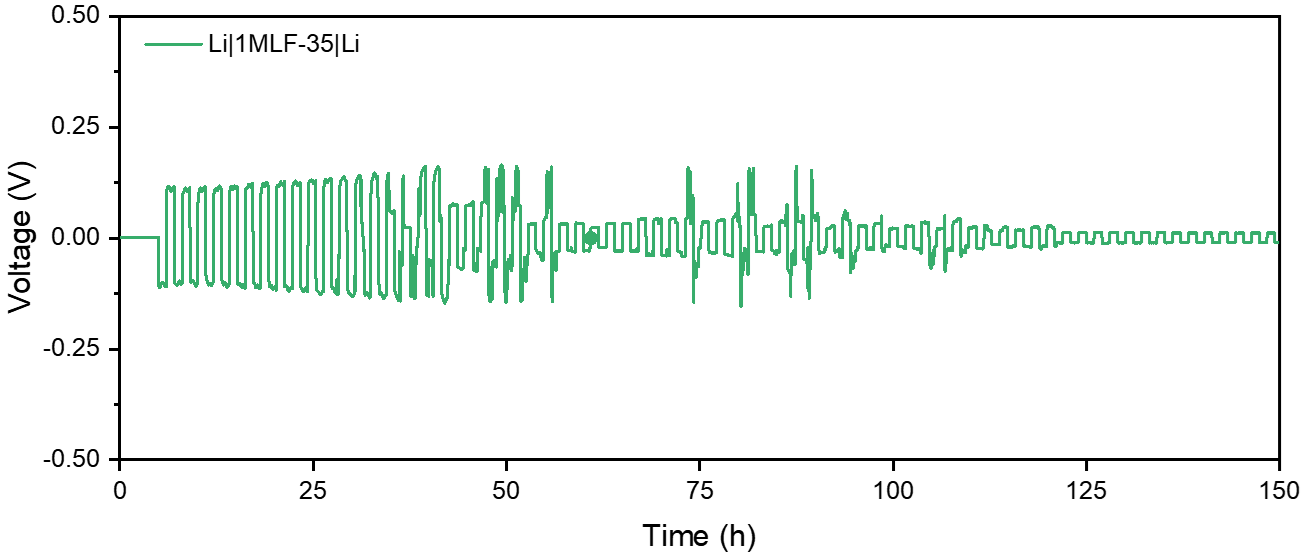


**Fig. S11** Long-term cycling of Li|1MLF-35|Li symmetrical cells, with a current density of 0.1 mA cm^-2^ and 0.05 mAh cm^-2^ Li plated and stripped per cycle


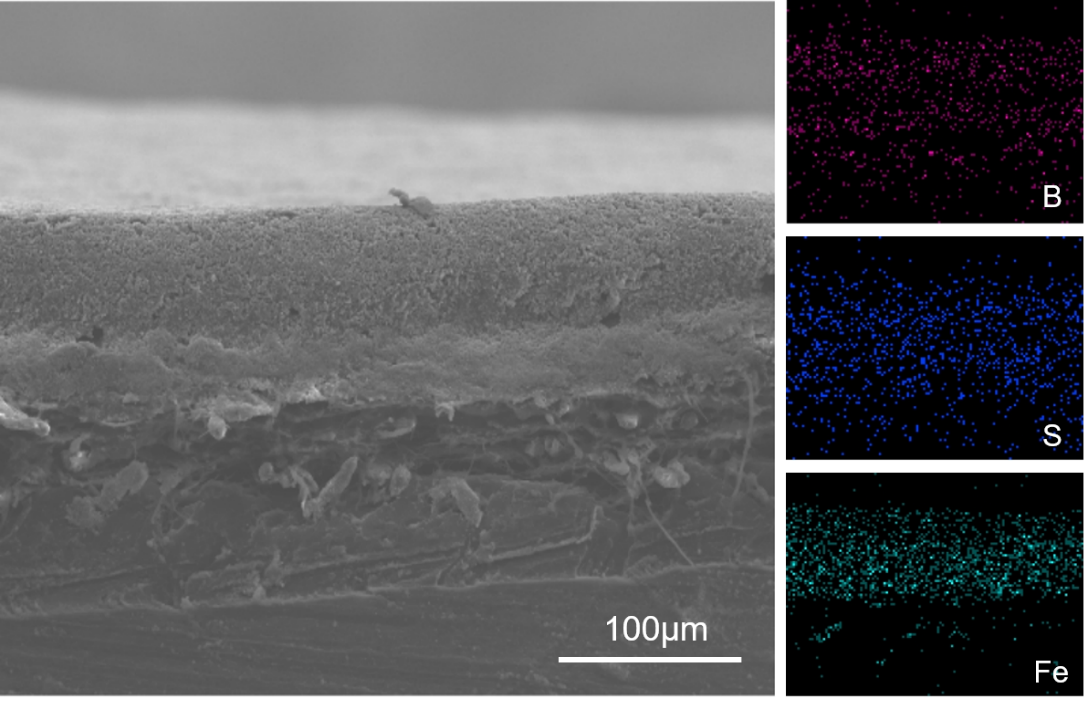


**Fig. S12** Cross-sectional SEM images and EDS mappings of the interface between SGPE and the LiFePO_4_ cathode

**Fig. S13** Typical charge–discharge curves of **a**) LiFePO_4_/SGPE/Li cell at 0.5 C, **b**) LiFePO_4_/LE/Li cell at 0.3 C, **c**) LiFePO_4_/CGPE/Li cell at 0.3 C and at room temperature


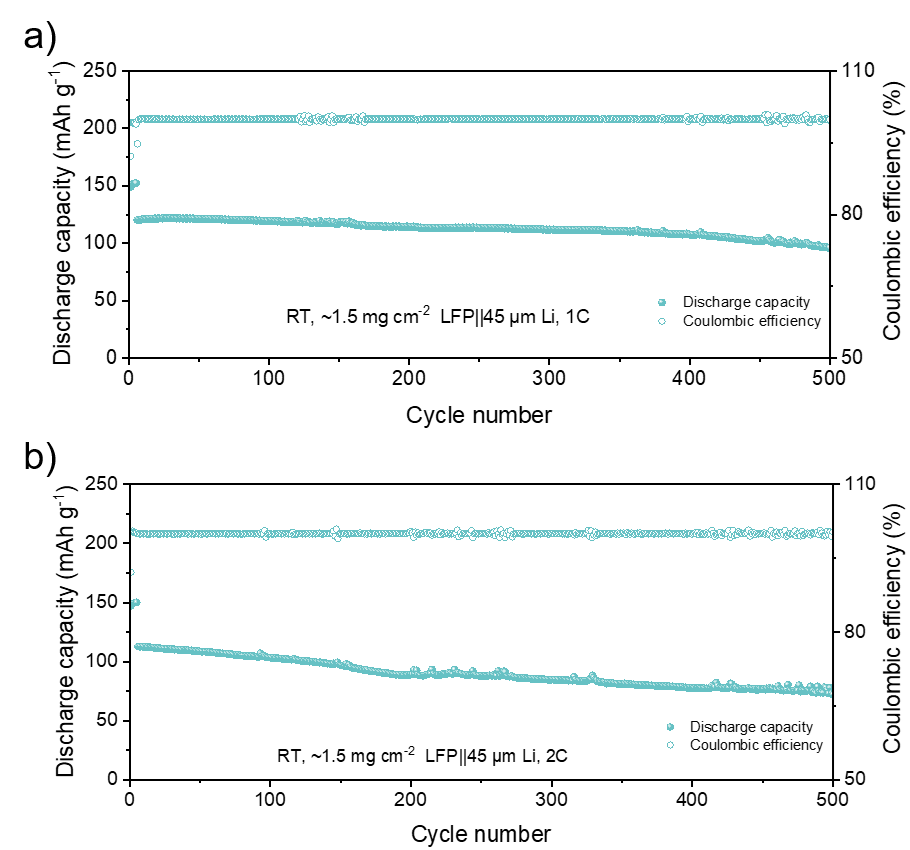


**Fig. S14** Long-term cycling performance for LiFePO_4_|SGPE|Li cell at a current density of (**a**) 1 C and (**b**) 2 C

**Fig. S15** Typical charge/discharge curves of **a**) LiFePO_4_/LE/Li cell, **b**) LiFePO_4_/CGPE/Li cell under the varied rate from 0.1 C to 2 C

**Fig. S16** Typical charge–discharge curves of a) LiCoO_2_|SGPE|Li cell, b) LiCoO_2_|LE|Li cell and c) LiCoO_2_|CGPE|Li cell at 0.2 C after being activated at 0.1 C at room temperature


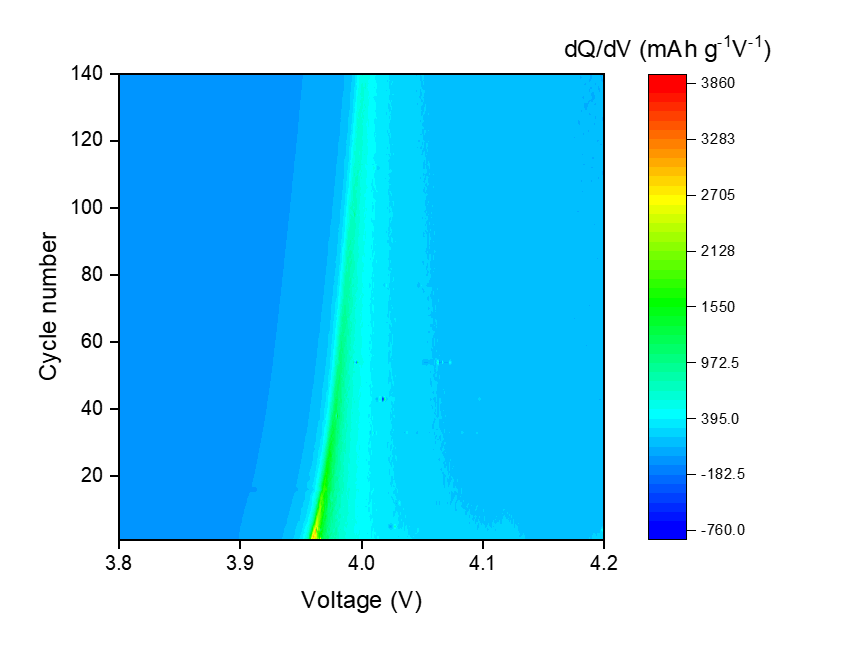


**Fig. S17** Contour plot of dQ/dV results of the LiCoO_2_|CGPE|Li cell from the 10th cycle to the 150th cycle

**
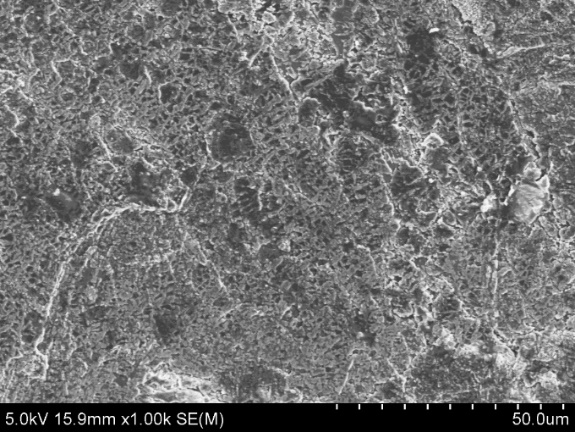

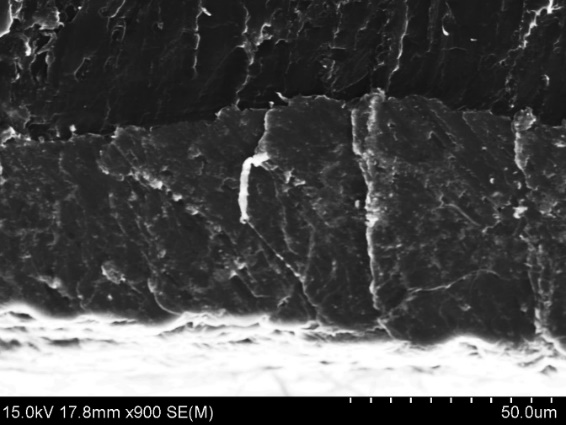
**

**
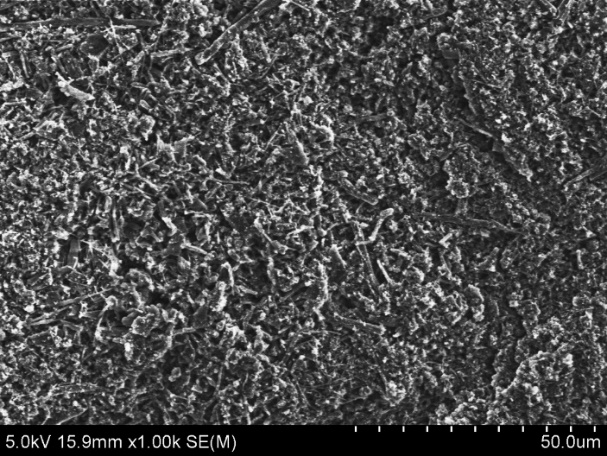

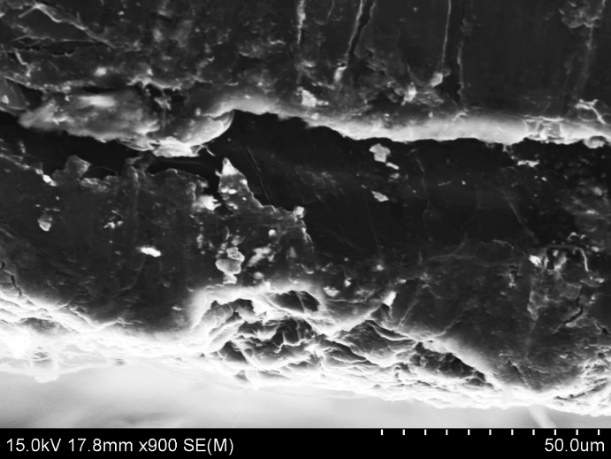
**

**Fig. S18** The raw SEM images of Fig. 5a-d, respectively


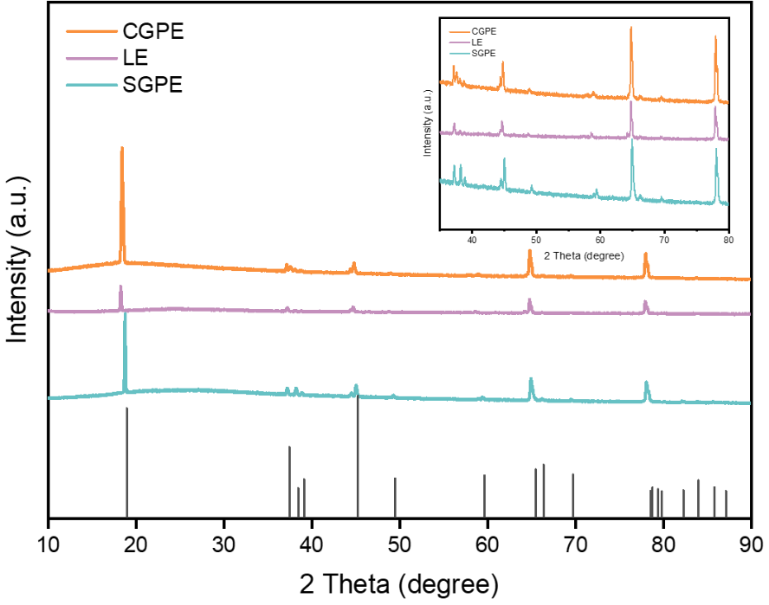


**Fig. S19** XRD patterns of pristine and cycled LiCoO_2_ cathodes after 30 cycles using SGPE, CGPE and LE electrolyte


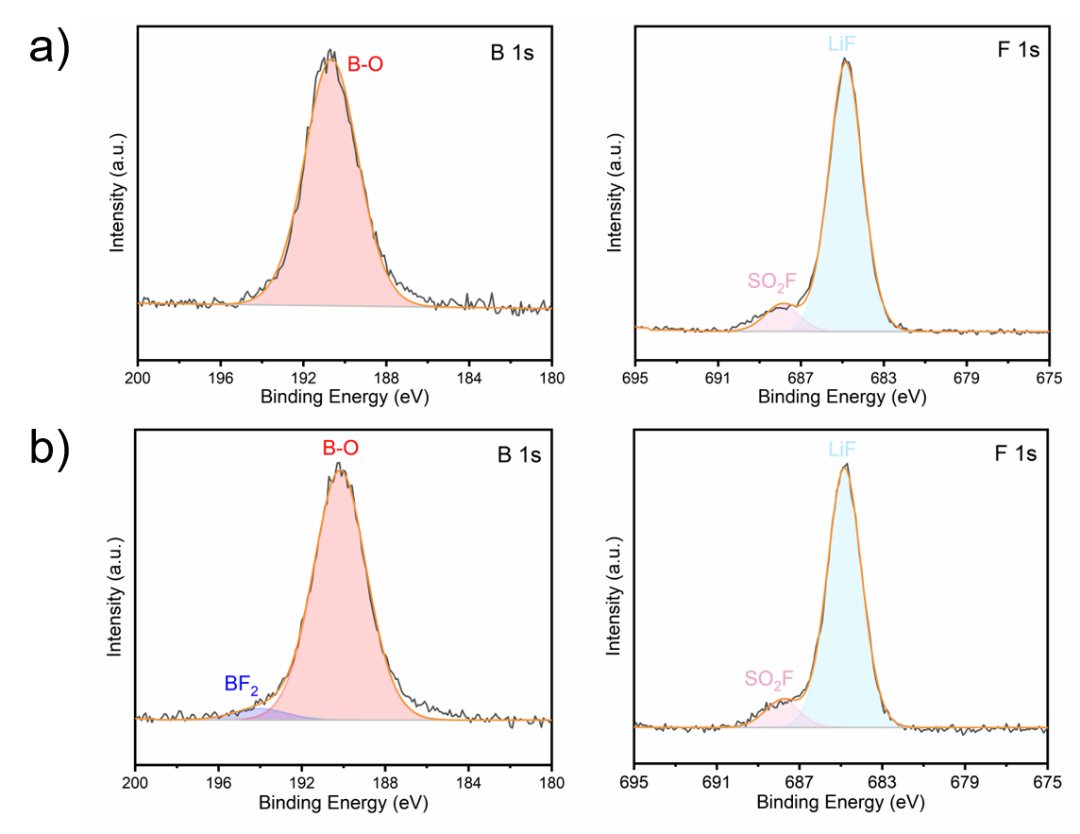


**Fig. S20** XPS spectra for B 1s and F 1s of the LiCoO_2_ cathodes cycled in **a**) SGPE electrolyte and **b**) CGPE electrolyte after 30 cycles


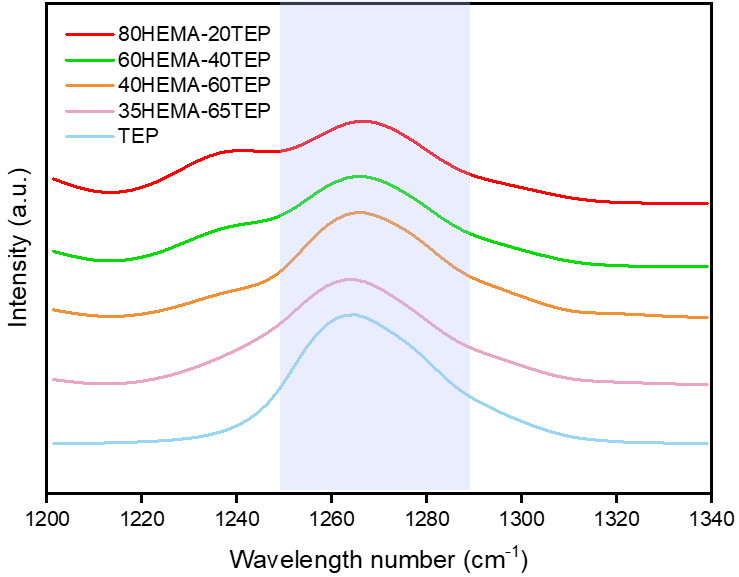


**Fig. S21** FTIR spectra of –P=O peaks in TEP-based samples with different HEMA concentrations

**Fig. S22** Deconvolution of peaks of the S-N-S stretching vibrational mode (CIP: contact ion pair, AGG: aggregated ion pair) in CGPE electrolyte

**Table S1** The compositions of electrolyte used to optimize ionic conductivity

| Electrolytes | LiTFSI  (mg) | TEP  (μL) | TFMA  (μL) | LiDFOB  (mg) | RT Ionic conductivity  (mS cm^-1^) |
| --- | --- | --- | --- | --- | --- |
| 35TFMA-0.2LF-0.5LB | 57.6 | 650 | 350 | 72 | 0.177 |
| 35TFMA-0.5LF-0.5LB | 144.0 | 650 | 350 | 72 | 0.933 |
| 35TFMA-1LF-0.5LB | 288.0 | 650 | 350 | 72 | 0.127 |
| 35TFMA-1.5LF-0.5LB | 432.0 | 650 | 350 | 72 | 0.070 |
| 35TFMA-2LF-0.5LB | 574.0 | 650 | 350 | 72 | 0.045 |
| 35TFMA-2.5LF-0.5LB | 718.0 | 650 | 350 | 72 | 0.021 |
| 40TFMA-0.5LF-0.5LB | 144.0 | 600 | 400 | 72 | 0.208 |
| 40TFMA-1.5LF-0.5LB | 432.0 | 600 | 400 | 72 | 0.041 |
| 10TFMA-0.5LF-0.5LB | 144.0 | 900 | 100 | 72 | 2.799 |
| 20TFMA-0.5LF-0.5LB | 144.0 | 800 | 200 | 72 | 1.495 |
| 30TFMA-0.5LF-0.5LB | 144.0 | 700 | 300 | 72 | 1.162 |
| 60TFMA-0.5LF-0.5LB | 144.0 | 400 | 600 | 72 | 0.009 |
| 60TFMA-1.5LF-0.5LB | 432.0 | 400 | 600 | 72 | 7.694E-04 |
| 80TFMA-0.5LF-0.5LB | 144.0 | 200 | 800 | 72 | 3.673E-06 |

**Table S2** Comparison in LiFePO_4_||Li full cells performance (upper voltage, final capacity, cycle numbers, C rate, running temperature and LFP loading) with the SGPE electrolyte and other reported electrolytes

| Type | Electrolyte | Anode  /Cathode | VR  (V) | Electrochemical  properties | Refs. | T | Loading  (mg/cm^2^) |
| --- | --- | --- | --- | --- | --- | --- | --- |
| Liquid electrolyte | 1 M LiTFSI  /EC/DEC | LiFePO4\|\|Li | 3.0-3.8 | 102.7 mAh g^-1^ for 125 cycles at 0.2 C, 65.2% capacity retention | [S1] | 25℃ | 2.08 |
| Gel electrolyte | PMLSE | LiFePO4\|\|Li | 3.0-3.8 | ~ 95 mAh g^-1^ for 200 cycles at 1 C,  85.5% capacity retention | [S1] | 25℃ | 2.08 |
| Gel electrolyte | PMLSE | LiFePO4\|\|Li | 3.0-3.8 | 127.7 mAh g^-1^ for 200 cycles at 0.2 C, 87.3% capacity retention | [S1] | 25℃ | 2.08 |
| Polymer electrolyte | PI/DBDPE  /PEO/LiTFSI | LiFePO4\|\|Li | 2.5-3.8 | ~120 mAh g^-1^ for 300 cycles at 0.5 C,  ~ 85% capacity retention | [S2] | 60℃ | 1.5 |
| Polymer electrolyte | PEO/LiTFSI | LiFePO4\|\|Li | 2.5-3.8 | ~100 mAh g^-1^ for 150 cycles at 0.5 C,  ~78% capacity retention | [S2] | 60℃ | 1.5 |
| Gel electrolyte | CMP/MMT | LiFePO4\|\|Li | 3.0-4.2 | 140 mAh g^-1^ for 400 cycles at 0.3 C,  >98% capacity retention | [S3] | RT | 5 |
| Gel electrolyte | CMP | LiFePO4\|\|Li | 3.0-4.2 | ~50 mAh g^-1^ for 400 cycles at 0.3 C,  rapid capacity decay | [S3] | RT | 5 |
| Hybrid SSE | LIM-L | LiFePO4\|\|Li | 2.5-4.2 | ~130 mAh g^-1^ for 150 cycles at 0.1 C;  97% capacity retention; 37 mAh g^−1^ at 0.8 C | [S4] | RT | 12.4 |
| Composite electrolyte | PVDFHFP/  LiFSI/LLZTO | LiFePO4\|\|Li | 2.5-4.0 | 142.2 mAh g^-1^ for 300 cycles at 0.5 C, 97.2% capacity retention | [S5] | RT | 2 |
| Gel electrolyte | PDIL | LiFePO4\|\|Li | 2.0-4.5 | 127.2 mAh g^-1^ for 50 cycles at 0.3 C, ~87.1% capacity retention | [S6] | 25℃ | 1.5 |
| Gel electrolyte | PDIL | LiFePO4\|\|Li | 2.0-4.5 | 111.1 mAh g^-1^ for 150 cycles at 1 C,  90.6% capacity retention | [S6] | 25℃ | 1.5 |
| Quasi-solid-state electrolyte | CLSPE-IL4 | LiFePO4\|\|Li | 2.5-4.0 | 136.7 mAh g^-1^ for 500 cycles at 0.2 C,  91% capacity retention | [S7] | 25℃ | 1.94 |
| Gel electrolyte | FRSE | LiFePO4\|\|Li | 2.5-4.0 | 147 mAh g^-1^ for 500 cycles at 0.5 C,  98.7% capacity retention | [S8] | RT | ~5.8 |
| Quasi-solid-state electrolyte | PEMD | LiFePO4\|\|Li | 2.5-3.9 | 155.6 mAh g^-1^ for 85 cycles at 0.2 C,  ~97% capacity retention | [S9] | 30 | 2.0-3.0 |
| Gel electrolyte | SGPE | LiFePO4\|\|Li | 2.5-4.0 | 110.4 mAh g^-1^ for 500 cycles at 0.5 C,  ~65.8% capacity retention | this work | RT | 1.5-2.0 |

**1 M LiTFSI/EC/DEC:** 1 M LiTFSI/EC/DEC, 1:1 by volume

**PMLSE:** PVDF-HFP/MOF composite gel/LLZN nanowires solid electrolyte

**PI:** polyimide

**DBDPE:** decabromodiphenyl ethane

**CMP:** a solid composite electrolyte, UV initiated in situ cross-linked of poly (ethylene glycol) methyl ether acrylate (MPEGA) and poly (ethylene glycol) diacrylate (PEGDA) between cathodes and anodes of cell

**CMP/MMT:** a novel solid polymer/montmorillonite (MMT) composite electrolyte

**LIM-L:** LI-IL@MOF-LLZO hybrid electrolyte

**PDIL:** polymer dispersed ionic liquid‐based solid polymer electrolyte (PDIL‐SPE)

**CLSPE-IL4:** the EA and VC based polymer electrolytes, with 40% mass ratio of [PY13] [TFSI], denoted as CLSPE-IL4

**FRSE:** a flame-retardant solid-liquid hybrid electrolyte

**PEMD:** a unique solid electrolyte complexion consisted of polyethylene oxide, UiO-66-NH_2_, and deep eutectic solvents

**Supplementary References**

1. J. Sun, X. Yao, Y. Li, Q. Zhang, C. Hou et al., Facilitating Interfacial Stability Via Bilayer Heterostructure Solid Electrolyte Toward High‐energy, Safe and Adaptable Lithium Batteries. Adv. Energy Mater. **10**(31), 2000709 (2020). <https://doi.org/10.1002/aenm.202000709>
2. Y. Cui, J. Wan, Y. Ye, K. Liu, L.-Y. Chou et al., A Fireproof, Lightweight, Polymer–Polymer Solid-State Electrolyte for Safe Lithium Batteries. Nano Lett. **20**(3), 1686–1692 (2020). <https://doi.org/10.1021/acs.nanolett.9b04815>
3. Y. Wang, X. Li, Y. Qin, D. Zhang, Z. Song et al., Local electric field effect of montmorillonite in solid polymer electrolytes for lithium metal batteries. Nano Energy **90**, 106490 (2021). <https://doi.org/10.1016/j.nanoen.2021.106490>
4. Z. Wang, Z. Wang, L. Yang, H. Wang, Y. Song et al., Boosting interfacial Li+ transport with a MOF-based ionic conductor for solid-state batteries. Nano Energy **49**, 580–587 (2018). <https://doi.org/10.1016/j.nanoen.2018.04.076>
5. J. Zhang, Y. Zeng, Q. Li, Z. Tang, D. Sun et al., Polymer-in-salt electrolyte enables ultrahigh ionic conductivity for advanced solid-state lithium metal batteries. Energy Storage Mater. **54**, 440–449 (2023). <https://doi.org/10.1016/j.ensm.2022.10.055>
6. S. Qin, Y. Cao, J. Zhang, Y. Ren, C. Sun et al., Polymer dispersed ionic liquid electrolytes with high ionic conductivity for ultrastable solid‐state lithium batteries. Carbon Energy **5**, e316 (2023). <https://doi.org/10.1002/cey2.316>
7. Z. Wang, Y. Wang, P. Zhai, P. Poldorn, S. Jungsuttiwong et al., A cation-dipole-reinforced elastic polymer electrolyte enabling long-cycling quasi-solid-state lithium metal batteries. J. Energy Chem. **75**, 340–348 (2022). <https://doi.org/10.1016/j.jechem.2022.08.042>
8. S.-J. Tan, J. Yue, Y.-F. Tian, Q. Ma, J. Wan et al., In-situ encapsulating flame-retardant phosphate into robust polymer matrix for safe and stable quasi-solid-state lithium metal batteries. Energy Storage Mater. **39**, 186–193 (2021). <https://doi.org/10.1016/j.ensm.2021.04.020>
9. S. Wang, Y. Chen, Q. Fang, J. Huang, X. Wang et al., Facilitating uniform lithium deposition via nanoconfinement of free amide molecules in solid electrolyte complexion for lithium metal batteries. Energy Storage Mater. **54**, 596–604 (2023). <https://doi.org/10.1016/j.ensm.2022.11.002>
